# Bayesian information sharing enhances detection of regulatory associations in rare cell types

**DOI:** 10.1093/bioinformatics/btab269

**Published:** 2021-07-12

**Authors:** Alexander P Wu, Jian Peng, Bonnie Berger, Hyunghoon Cho

**Affiliations:** Computer Science and Artificial Intelligence Laboratory, MIT, Cambridge, MA 02139, USA; Department of Computer Science, University of Illinois at Urbana-Champaign, Champaign, IL 61801, USA; Computer Science and Artificial Intelligence Laboratory, MIT, Cambridge, MA 02139, USA; Department of Mathematics, MIT, Cambridge, MA 02139, USA; Broad Institute of MIT and Harvard, Cambridge, MA 02142, USA; Broad Institute of MIT and Harvard, Cambridge, MA 02142, USA

## Abstract

**Motivation:**

Recent advances in single-cell RNA-sequencing (scRNA-seq) technologies promise to enable the study of gene regulatory associations at unprecedented resolution in diverse cellular contexts. However, identifying unique regulatory associations observed only in specific cell types or conditions remains a key challenge; this is particularly so for rare transcriptional states whose sample sizes are too small for existing gene regulatory network inference methods to be effective.

**Results:**

We present ShareNet, a Bayesian framework for boosting the accuracy of cell type-specific gene regulatory networks by propagating information across related cell types via an information sharing structure that is adaptively optimized for a given single-cell dataset. The techniques we introduce can be used with a range of general network inference algorithms to enhance the output for each cell type. We demonstrate the enhanced accuracy of our approach on three benchmark scRNA-seq datasets. We find that our inferred cell type-specific networks also uncover key changes in gene associations that underpin the complex rewiring of regulatory networks across cell types, tissues and dynamic biological processes. Our work presents a path toward extracting deeper insights about cell type-specific gene regulation in the rapidly growing compendium of scRNA-seq datasets.

**Supplementary information:**

Supplementary data are available at *Bioinformatics* online.

**Availability and implementation:**

The code for ShareNet is available at http://sharenet.csail.mit.edu and https://github.com/alexw16/sharenet.

## 1 Introduction

Understanding the intricate coordination of biomolecules underlying transcriptional regulation, commonly represented as a gene regulatory network (GRN), is key to gaining mechanistic insights into the diversity of cellular functions and phenotypes observed across different cell types and biological states. Single-cell RNA sequencing (scRNA-seq) (Gawad *et al.*, 2016) has emerged as a powerful way of profiling the transcriptomes of individual cells, rather than the average of large populations of cells as in traditional bulk sequencing. scRNA-seq has already played a pivotal role in uncovering the rich heterogeneity of gene expression patterns (Hie *et al.*, 2020), enabling the decomposition of diverse populations of cells into their component cell types (Bjorklund *et al.*, 2016; Buettner *et al.*, 2015; Hie *et al.*, 2019a) as well as *de novo* identification of rare cell types (DeMeo and Berger, 2020; Grün *et al.*, 2015; Hie *et al.*, 2019b; Jiang *et al.*, 2016; Villani *et al.*, 2017). We reasoned that scRNA-seq could also enhance GRN inference by allowing for more accurate assessment of covarying patterns among genes. The rapid growth of scRNA-seq datasets spanning diverse tissues, organisms and conditions thus provides an enticing opportunity for studying gene regulatory interactions at an unprecedented scale and resolution.

**Fig. 1.**
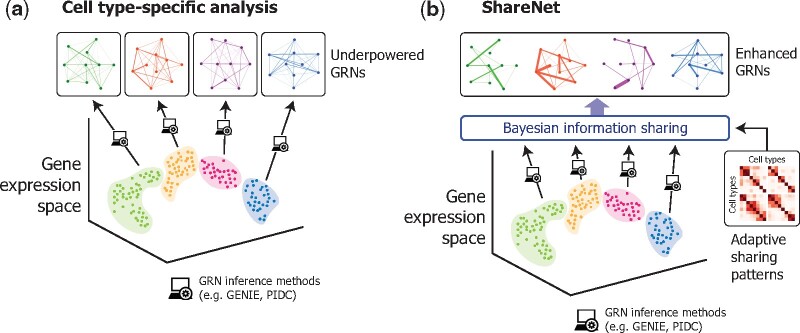
Overview of ShareNet framework. (**a**) Analyzing gene regulatory interactions in individual cell types is challenging when only a small number of cells for a given cell type have been profiled in a single-cell RNA sequencing experiment. (**b**) ShareNet shares information across cell types through a Bayesian framework to enhance the accuracy of predicted regulatory interactions in each cell type. ShareNet adaptively chooses the information sharing structure that best explains the input data. GRN: gene regulatory network

Despite the large success of cell type identification analyses based on scRNA-seq (Bjorklund *et al.*, 2016; Buettner *et al.*, 2015; Grün *et al.*, 2015; Jiang *et al.*, 2016), relatively few efforts have been made to further characterize the cell types by inferring their underlying GRNs. Although numerous methods for general GRN reconstruction exist in the literature, including state-of-the-art tools such as GENIE3 (Huynh-Thu *et al.*, 2010) and PIDC (Chan *et al.*, 2017), a key challenge in applying these methods in a cell type-specific context has been the severe lack of power when analyzing rare or sparsely sampled cell types (Fig. 1a). This problem is further compounded by the inherent sparsity of scRNA-seq data stemming from both technical and biological factors (Angerer *et al.*, 2017; Choi *et al.*, 2020), which naturally increases the number of samples needed for accurate inference. Although increasing the number of sequenced cells in a study partially mitigates this issue by obtaining more cells from rare cell types, larger datasets also tend to reveal additional rare cell types to be studied as well as fine-grain transcriptional structure *within* a known cell type; both may still suffer from similar sample size limitations, not to mention the burden of increased experimental cost of sequencing more cells. Since the detection and characterization of rare cell types is a prominent goal for many single-cell studies (Grün *et al.*, 2015; Jiang *et al.*, 2016; Villani *et al.*, 2017), these issues present a major hurdle in understanding the regulatory patterns of rare, yet important transcriptional states.

We introduce ShareNet, a Bayesian information sharing framework for increasing the accuracy of predicting cell type-specific regulatory associations from single-cell transcriptomic data (Fig. 1b). Our framework draws upon the intuition that many of the regulatory interactions (and non-interactions) are shared across different cell types, due to shared developmental lineages, regulatory programs, or biophysical constraints. Thus, by propagating information across related cell types, we hope to reduce noise and boost the accuracy of inferred GRNs in all cell types. Since we do not have full knowledge of the sharing patterns underlying a given dataset, we designed our framework to adaptively learn a multifactorial, information sharing structure that best explains the data in all the study’s cell types. Importantly, our framework is widely applicable, as it can serve as an additional layer on top of existing state-of-the-art network inference algorithms to enhance their accuracy in estimating the GRNs of all cell types in a dataset.

In our work, we demonstrate the effectiveness of ShareNet on a range of scRNA-seq datasets at boosting the accuracy of GENIE3 (Huynh-Thu *et al.*, 2010) and PIDC (Chan *et al.*, 2017), two of the top-performing algorithms from a recent single-cell network inference benchmarking study (Pratapa *et al.*, 2020), as well as standard co-expression networks based on Pearson correlation. We also show that ShareNet uncovers cell type-specific gene associations that uniquely characterize the identity of cells within different tissue contexts and across differentiation trajectories.

As scRNA-seq datasets continue to uncover new rare cell types and transcriptional states, the ability of ShareNet to accurately determine higher-order transcriptional patterns in small subpopulations of cells in a dataset will be increasingly crucial for scientific studies. An open-source implementation of ShareNet is available at: https://github.com/alexw16/sharenet.

## 2 Materials and methods

### 2.1 Overview of ShareNet

ShareNet produces improved estimates of regulatory associations by leveraging both the redundancy and diversity of gene regulatory network structures underlying the many transcriptional states observed in a single-cell RNA-seq dataset. At its core is a compact set of cell type-to-cell type sharing patterns that is learned directly from the data. This set of sharing patterns determines the manner by which each edge in a network for a specific cell type propagates information about its presence or absence to the same edge in each of the other cell types’ networks. By learning a collection of sharing patterns rather than a single common pattern, ShareNet is able to adaptively share information across cell types in a manner that spans a broad range of possible cell type-to-cell type relationships rather than being limited to a singular sharing structure. The flexibility offered by this approach is especially useful for inferring networks, as each edge may be specific to a particular cell type, shared across a subset of related cell types or globally shared with all cell types depending on the scope of biological functions with which the edge is associated. Intuitively, ShareNet automatically learns and leverages the specific sharing pattern that is most appropriate for each edge. Furthermore, ShareNet is compatible with a broad range of existing network inference algorithms to boost their accuracy in constructing cell type-specific networks.

### 2.2 ShareNet’s Bayesian model for information sharing

ShareNet minimally requires that the chosen algorithm outputs a continuous score for each putative edge of interest in a network for each cell type. A user would first apply his/her network inference algorithm of choice to each cell type in a dataset to generate an initial estimate of the edge scores for each cell type. ShareNet models these initial network estimates as noisy observations of true underlying edge scores, governed by a hierarchical Bayesian model that induces similarities in predictions between similar cell types.

In this Bayesian model, the collection of latent cell type-to-cell type information sharing patterns is represented as a mixture of multivariate Gaussian distributions with *K* mixture components.

(1) $$u_{i,j}∼\text{Categorical}(1/K,\ldots ,1/K)$$

 

(2) $$z_{i,j}|u_{i,j}∼N(\mu_{u_{i,j}},\Sigma_{u_{i,j}})$$

Here, $z_{i,j}$ is a *C*-dimensional vector, where *C* is the number of cell types in a dataset. Each of the *C*-by-*C* covariance matrices $\Sigma_{1},\ldots ,\Sigma_{K}$ represents a unique cell type-to-cell type sharing pattern, with the off-diagonal entries in each covariance matrix capturing potential positive or negative correlations in the predicted edge scores between cell types.

For regularization, we place a Normal-Wishart (NW) prior over each of the mean $\mu_{k}$ and the inverse covariance $\Sigma_{k}^{-1}$ parameters belonging to the *K* sharing patterns.

(3) $$\left(\mu_{k},\Sigma_{k}^{-1})∼\text{NW}(\mu_{0},\beta_{0},\Psi ,\nu \right)$$

Each element of the *C*-dimensional vector $z_{i,j}=(z_{i,j}^{\left(1\right)},\ldots ,z_{i,j}^{\left(C\right)})$ then serves as a parameter for a univariate Gaussian distribution that describes the noisy distribution of an edge’s score from the chosen network inference algorithm.

(4) $$e_{i,j}^{\left(c\right)}∼N(z_{i,j}^{\left(c\right)},\sigma_{i,j}^{(c)2})$$

Here, $e_{i,j}^{\left(c\right)}$ represents the observed score for the edge connecting gene *i* to gene *j* in cell type *c*. In this setup, the mean $z_{i,j}^{\left(c\right)}$ of the Gaussian is a latent variable that represents the true score of the edge in an ideal scenario absent of noise. Note that the noise distribution for each edge score is assumed to be Gaussian, a condition that is approximately satisfied by a range of methods we consider in this work (Supplementary Fig.  S1). Optionally, one can model the mean of each edge distribution as $g(z_{i,j}^{\left(c\right)})$, where g(·) describes a link function that provides further flexibility for modeling the sharing patterns of edge scores; all of our results are based on the default configuration without this added non-linearity.

The variance parameter $\sigma_{i,j}^{(c)2}$ captures the degree of variation in the observed edge score. Importantly, the variation captured by $\sigma_{i,j}^{(c)2}$ represents the aggregate set of biological, technical and sample size factors that may contribute to noisy estimates of the edge score. A more detailed discussion of this variance term is presented in the next section.

### 2.3 Bootstrap estimation of edge-specific noise distribution

The task of inferring cell type-specific gene regulatory networks from single-cell transcriptomics data is challenging due to a wide array of biological, technical and sample size factors. In our probabilistic model, we characterize the cumulative effect of these factors by estimating the standard deviation for each observed edge score in a network with respect to the network inference algorithm of choice. We empirically estimate the standard deviation by (i) generating a bootstrap sample of cells for a particular cell type *c* that contains the same number of cells as all of the cells in that cell type, (ii) applying the network inference algorithm of choice for the bootstrap sampled set of cells and (iii) repeating (i) and (ii) across *M* trials. This process generates *M* estimates of the network for cell type *c*. We then calculate the sample standard deviation for each edge score across the *M* trials. We denote this quantity as ${\hat{\sigma }}_{i,j}^{\left(c\right)}$ for edge (*i*, *j*) in cell type *c*. We use these terms in the last layer of our model as an estimate for the standard deviation of the edge distributions. More precisely, we set the $\sigma_{i,j}^{\left(c\right)}$ term in the Gaussian distribution $N(e_{i,j}^{\left(c\right)}|z_{i,j}^{\left(c\right)},\sigma_{i,j}^{(c)2})$, from which the edge score $e_{i,j}^{\left(c\right)}$ is drawn, to be equal to ${\hat{\sigma }}_{i,j}^{\left(c\right)}$. Networks inferred for smaller cell type populations display higher edge score standard deviations across various scRNA-seq datasets and network inference algorithms (Supplementary Fig.  S2), suggesting that inferring networks using existing techniques is indeed underpowered for rare cell types.

### 2.4 End-to-end generative model for likelihood analysis

Although ShareNet models only the predicted edge scores from the network inference method of choice, we sought to further extend the ShareNet model to obtain an end-to-end generative process for calculating the likelihood of observed gene expression levels. We use this full model, which we refer to as BVS (Bayesian variable selection), for holdout likelihood analyses in our benchmark experiments to provide additional evidence for the utility of information sharing. To this end, we frame the task of network inference as a variable selection problem, in which the goal is to identify putative regulators for target genes. In particular, we adapt a Bayesian approach proposed by Carbonetto and Stephens (2012) for identifying causal variants in genetic association studies and integrate ShareNet as a prior for variable selection.

We denote the scRNA-seq expression values for a set of regulator genes for cell type *c* as $X^{\left(c\right)}$. The expression of the corresponding target genes $y^{\left(c\right)}$ is modeled as a linear combination of the putative regulators plus Gaussian noise:

(5) $$y_{j,n}^{\left(c\right)}∼N(\sum_{i}X_{i,n}^{\left(c\right)}\beta_{i,j}^{\left(c\right)}\gamma_{i,j}^{\left(c\right)},\sigma_{\epsilon }^{2}),$$

where $\beta_{i,j}^{\left(c\right)}$ represents the coefficient of the linear model describing the effect of regulator gene *i* on target gene *j*, and $\gamma_{i,j}^{\left(c\right)}$ is a corresponding binary indicator variable that indicates whether or not regulator gene *i* is to be included in the network model for cell type *c*. We also include the following ‘spike and slab’ priors (Mitchell and Beauchamp, 1988) on $\beta_{i,j}^{\left(c\right)}$ and $\gamma_{i,j}^{\left(c\right)}$.

(6) $$\gamma_{i,j}^{\left(c\right)}∼\text{Bernoulli}(g(z_{i,j}^{\left(c\right)}))$$

 

(7) $$\beta_{i,j}^{\left(c\right)}|\gamma_{i,j}^{\left(c\right)}∼\{\begin{array}{cc} N(0,\sigma_{\beta }^{2}) & \;\text{if}\;\gamma_{i,j}^{\left(c\right)}=1,\\ \delta_{0}(\beta_{i,j}^{\left(c\right)}) & \;\text{otherwise},\; \end{array}$$

where $\delta_{0}(\cdot )$ denotes the Dirac delta function. Here, $z_{i,j}^{\left(c\right)}$, like in the previous section, is modeled as a draw from the mixture of multivariate Gaussians describing the set of cell type-to-cell type sharing patterns we seek to leverage in ShareNet. We include the sigmoid function $g(x)=1/(1+e^{-x})$ in this setting in order to map the Gaussian to a value between 0 and 1 for appropriate use as a Bernoulli parameter. In the scenario where there is no information sharing, the prior over $\gamma_{i,j}^{\left(c\right)}$ is simply a Bernoulli distribution with fixed hyperparameter *π*.

### 2.5 Estimating posterior beliefs using variational inference

Given our model, re-estimating the edge scores based on information sharing reduces to calculating the posterior density of the latent variables given the input data. Although exact inference is intractable, we can leverage variational inference to approximately solve this inference task (Blei *et al.*, 2017). Briefly, the standard variational inference approach involves reframing our problem of computing the posterior as an optimization problem, in which we first propose a family of variational distributions for approximating the true posterior. We then identify the distribution within this family that most closely resembles the posterior, using KL divergence as the optimization objective.

(8) $$q^{*}(U)=\underset{q}{\arg\min}\;\text{KL}(q(U)||p(U|e))$$

Here, *p* denotes the true model distribution, *q* denotes the variational distribution and ***U*** denotes the set of latent variables in our model whose posterior distributions we aim to approximate. In the ShareNet model, we have $U=(\mu ,\Sigma^{-1},u,z)$. Following standard techniques, we assume that the variational distribution factorizes as

(9) $$q(\mu ,\Sigma^{-1},u,z)=\prod_{k=1}^{K}q(\mu_{k},\Sigma_{k}^{-1})\prod_{n=1}^{N}q(z_{n})q(u_{n}),$$

where each subscript for $u_{n}$ and $e_{n}$ maps to a unique edge (*i*, *j*). In addition, we restrict each of the factors in our variational distribution to take on the following distributions parameterized by the accompanying variational parameters.

(10) $$q(\mu_{k},\Sigma_{k}^{-1})=\text{NW}(\mu_{k},\Sigma_{k}^{-1}|{\tilde{a}}_{k},{\tilde{\beta }}_{0},{\tilde{B}}_{k},{\tilde{\nu }}_{k})$$

 

(11) $$q(z_{n})=N(z_{n}|{\tilde{m}}_{n},{\tilde{S}}_{n})$$

 

(12) $$q(u_{n})=\text{Categorical}({\tilde{\varphi }}_{n})$$

For BVS, we include the variables that define the linear model, so $U=(\mu ,\Sigma^{-1},u,z,\beta ,\gamma )$, and the corresponding model factorizes as

(13) $$q(\mu ,\Sigma^{-1},u,z,\beta ,\gamma )=\prod_{k=1}^{K}q(\mu_{k},\Sigma_{k}^{-1})\prod_{n=1}^{N}q(z_{n})q(u_{n})q(\beta_{n},\gamma_{n}),$$

where $q(\beta_{n},\gamma_{n})=\prod_{c=1}^{C}q(\beta_{n}^{\left(c\right)},\gamma_{n}^{\left(c\right)})$. We parameterize the variational distributions for this approach in a similar manner as above and also include the following distribution.

(14) $$q(\beta_{n}^{\left(c\right)}|\gamma_{n}^{\left(c\right)})=\{\begin{array}{cc} {\tilde{\alpha }}_{n}^{\left(c\right)}N(\beta_{n}^{\left(c\right)}|{\tilde{v}}_{n}^{\left(c\right)},{\tilde{s}}_{n}^{(c)2}) & \;\text{if}\;\gamma_{n}^{\left(c\right)}=1,\\ \left(1-{\tilde{\alpha }}_{n}^{\left(c\right)})\delta_{0}(\beta_{i,j}^{\left(c\right)}\right) & \text{otherwise}. \end{array}$$

### 2.6 Coordinate ascent optimization algorithm

In seeking to identify the variational distribution that best matches the posterior, we minimize the KL divergence objective equivalently by maximizing the ELBO (evidence lower bound), with respect to the variational parameters. The ELBO objective can be expressed as follows.

(15) $$\text{ELBO}(q)=E_{q}[\text{log}\;p(\mu ,\Sigma ,u,z,e)]-E_{q}[\text{log}\;q(\mu ,\Sigma ,u,z)]$$

We optimize this quantity by coordinate ascent, which entails setting the partial derivative of the ELBO with respect to each variational parameter to zero and then solving for the conditionally optimal update for each variational parameter. The resulting update equations and the full derivation are provided in Supplementary Materials.

Finally, to extract the enhanced networks from our model, we leverage the variational distributions to approximate key latent variables of interest. Specifically, note that ${\tilde{m}}_{i,j}^{\left(c\right)}$ approximates the posterior mean of $z_{i,j}^{\left(c\right)}$, which represents the true edge score for a chosen network inference algorithm. Hence, we use the learned ${\tilde{m}}_{i,j}^{\left(c\right)}$ values as revised edge scores.

### 2.7 Datasets and pre-processing

We analyzed three scRNA-seq datasets that we refer to as the BEELINE (Pratapa *et al.*, 2020), Tabula muris (Schaum *et al*., 2018) and mouse blood lineage datasets (Pijuan-Sala *et al.*, 2019). The pre-processed BEELINE dataset was made available online by the authors, and we made no further modifications to this dataset. For the Tabula muris and mouse blood lineage datasets, we first applied a standard log(1+x/100) transformation to the raw counts data (provided in counts per million [CPM]) and removed genes that were not expressed in at least 10% of cells in any cell type. In inferring networks, we considered the set of genes defined by the union of the set of genes that are annotated as transcription factors in the AnimalTFDB database (Hu *et al.*, 2019) and the set of 1000 genes with the highest dispersion for each dataset (Satija *et al.*, 2015).

In order to assess the accuracy of networks inferred using ShareNet, we compared networks estimated with and without ShareNet to a collection of reference networks derived from a range of existing molecular interaction databases. This collection of reference networks included (i) a functional interaction network derived from the STRING database (Szklarczyk *et al.*, 2018), (ii) a non-specific ChIP-seq network constructed using ChIP-seq data from the DorothEA (Garcia-Alonso *et al.*, 2019), RegNetwork (Liu *et al.*, 2015) and TRRUST (Han *et al.*, 2018) databases and (iii) multiple cell type-specific ChIP-seq networks derived from ChIP-seq data matched to each of the specific cell types represented in the scRNA-seq dataset that we analyzed. For studying networks inferred from the BEELINE scRNA-seq datasets, the cell type-specific ChIP-seq networks used as reference were built using data from the ENCODE (ENCODE Project Consortium, 2012), ChIP-Atlas (Oki *et al.*, 2018) and ESCAPE (Xu *et al.*, 2014) databases. This set of STRING, non-specific ChIP-seq and cell type-specific ChIP-seq reference networks were obtained from the BEELINE benchmarking study (Pratapa *et al.*, 2020). For the remaining scRNA-seq datasets that we analyzed, the cell type-specific ChIP-seq networks were obtained from ChIP-Atlas (Oki *et al.*, 2018), and an edge was defined for a transcription factor-target gene pair if a ChIP peak called using MACS2 (Zhang *et al.*, 2008) ($q<10^{-5}$) for the transcription factor was located within 5 kb of the target gene’s transcription start site.

### 2.8 Evaluation strategies

For each inferred network, we computed the area under the precision-recall curve (AUPRC) relative to each of these three sets of reference networks to quantify the agreement between the predicted network and these reference networks. Similar to the BEELINE benchmarking study, when comparing inferred networks to either of the non-specific or specific ChIP-seq networks, we only considered edges outgoing from transcription factors for which ChIP-seq data was available.

In the case of our extended BVS model described in Section 2.4, we are also able to evaluate the ability of the model to accurately predict gene expression values for a network’s target genes. The linear predictor in BVS defines the relationship between each target gene and a set of regulator genes, and we evaluated the effectiveness of this predictor on unseen data by calculating the model’s posterior predictive distribution with respect to held out data. To this end, we split each cell type in a dataset into a training set comprising 80% of cells in that cell type and held out the remaining 20% of cells as a test. We trained our model on the training set and then calculated a test log likelihood value for each target gene based on the posterior predictive distribution.

### 2.9 Hyperparameter selection

The use of ShareNet involves the consideration of two hyperparameters, the number of mixture components and the number of bootstrapped samples used to estimate edge score uncertainties. While the number of mixture components can be automatically learned via a nested cross-validation procedure, we found that ShareNet’s performance is generally robust to the number of mixture components, as long as multiple mixture components are used (Supplementary Fig. S3). We also determined that the standard deviation estimates for a majority of edges converge in value upon approximately five bootstrapped samples across Pearson correlation, GENIE3 and PIDC (Supplementary Fig. S4). For the below experiments, we used ten mixture components in our model and five bootstrapped samples for calculating edge score uncertainties.

## 3 Results

### 3.1 ShareNet improves network inference accuracy on a range of scRNA-seq datasets

#### 3.1.1 BEELINE benchmark single-cell data

We first evaluated ShareNet on a set of well-defined scRNA-seq datasets introduced in a recent study (Pratapa *et al.*, 2020) to benchmark the state-of-the-art network inference algorithms. The evaluation framework used in this study, called BEELINE, leveraged data from five broad categories of mouse cell types: embryonic stem cells, dendritic cells, erythroid cells, granulocyte-monocyte cells and lymphoid cells. We asked whether one could exploit any similarity in the network structures underlying these five cell types to simultaneously improve the accuracy of these networks. To that end, we demonstrated the applicability of ShareNet on three common network inference approaches: GENIE3 (Huynh-Thu *et al.*, 2010), PIDC (Chan *et al.*, 2017) and Pearson correlation.

We sought to evaluate the effectiveness of ShareNet in improving the accuracy of networks inferred in small sample size settings. To do so, we simulated various small sample size settings in the BEELINE reference datasets by downsampling the population of dendritic cells to population sizes of 50, 100 and 200 cells, while the other four cell types’ populations were retained in full. For each of the 50, 100 and 200 cell type population sizes, we inferred networks for dendritic cells and for the remaining cell types with and without ShareNet across the various network inference approaches.

**Fig. 2.**
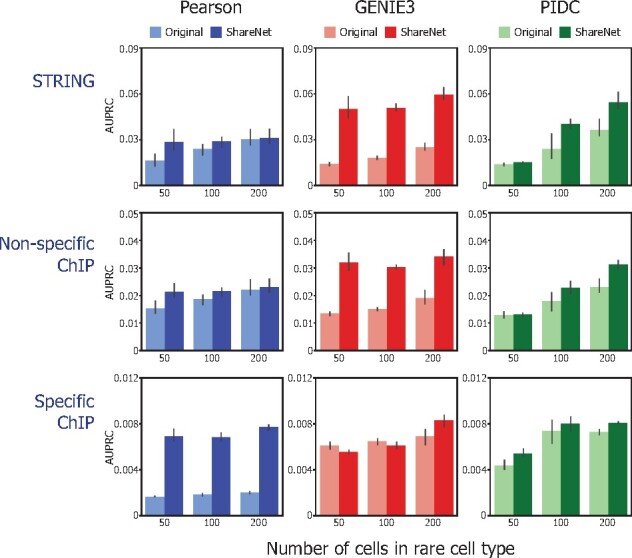
ShareNet improves network inference accuracy for small populations of dendritic cells in BEELINE benchmark single-cell RNA-seq datasets. We compared the accuracy of dendritic cell networks generated based on Pearson correlation, GENIE3 and PIDC, with and without ShareNet. Dendritic cell population is downsampled to 50, 100 and 200 cells to assess the effect of undersampling. Inferred networks are compared with reference networks obtained from STRING, non-cell type-specific ChiP-seq data and cell type-specific ChIP-seq data. Overall, ShareNet-augmented methods result in better agreement with reference networks as compared to the original methods

Across all three network inference algorithms, we observed that the inclusion of ShareNet produced networks that achieved higher AUPRC overall compared to the corresponding networks inferred in the setting without ShareNet (Fig. 2). This improvement in performance was most notable for the scenarios where dendritic cells were downsampled most aggressively. For the 50 cell population size setting, the three network inference algorithms predictably demonstrated worse performance compared to in the larger 100 and 200 cell population size settings. However, when used with ShareNet, the accuracy of networks inferred for the 50 cell population of dendritic cells not only consistently improved but in several instances also exceeded the accuracy of the networks inferred using 100 and 200 cells without ShareNet. This outcome was observed across both the STRING and non-specific ChIP-seq as well as the cell type-specific ChIP-seq networks. The only exception was the slightly decreased accuracy of networks inferred using GENIE3 for cell type population sizes of 50 and 100 when compared with the cell type-specific ChIP-seq network. We hypothesize that this is due to the considerably greater variability of bootstrapped GENIE3 edge scores for these smallest populations of cells (Supplementary Fig. S5). This greater uncertainty increases the degree of information sharing across cell types, thus explaining the simultaneous decrease of accuracy with respect to the cell type-specific ChIP-seq network and increase of accuracy with respect to the STRING and non-specific ChIP-seq networks for these smaller cell type populations.

Overall, these results suggest that ShareNet propagates information across cell types in a manner that benefits the discovery of both cell type-specific and non-specific regulatory associations. Furthermore, even though the remaining four cell types’ populations were not downsampled and were represented by larger numbers of cells, their respective networks also saw increases in accuracy in some cases while consistently avoiding notable deterioration in performance from sharing information with the downsampled dendritic cells (Supplementary Fig. S6).

#### 3.1.2 Tabula Muris and mouse blood lineage datasets

To demonstrate ShareNet on a broad range of use cases for single-cell experiments, we tested on two other scRNA-seq datasets: (i) the Tabula Muris dataset containing an extensive collection of cell types isolated from 20 mouse organs and tissues (Schaum *et al*., 2018) and (ii) a mouse blood lineage dataset consisting of cells along a differentiation trajectory in early developmental stages that includes rare transitional states (Pijuan-Sala *et al.*, 2019).

Each cell in these datasets was previously assigned cell type annotations based on known markers and genes, and we sought to infer networks for the groups of cells corresponding to these annotations. We inferred networks for each cell type across the three different datasets using GENIE3, PIDC and Pearson correlation. Similar to the benchmarking analyses performed on the BEELINE dataset, we used a variety of reference networks to assess the accuracy of the inferred networks. We used the aforementioned STRING functional interaction network and the non-specific ChIP-seq networks in our analysis. In order to determine the accuracy of the inferred networks in detecting cell type-specific regulatory interactions, we also obtained ChIP-seq data from ChIP-Atlas (Oki *et al.*, 2018) for as many of the specific cell types identified in the Tabula Muris and mouse blood lineage datasets as were available.

**Fig. 3.**
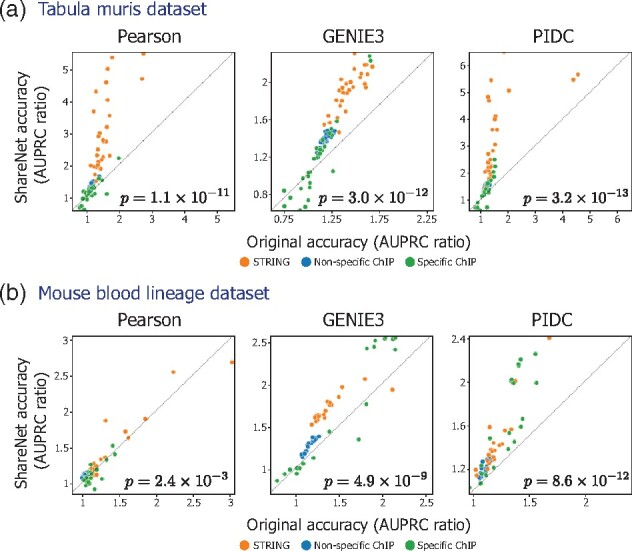
ShareNet improves network inference accuracy for (**a**) Tabula Muris and (**b**) mouse blood lineage single-cell RNA-seq datasets. We compared the accuracy of networks generated based on Pearson correlation, GENIE3 and PIDC with (*y*-axis) and without (*x*-axis) ShareNet. Different reference networks used for evaluation are marked by different colors. We plot the ratio of the AUPRC metric relative to the AUPRC associated with a random predictor to map the results from different reference networks onto a common scale. *P*-values indicate the statistical significance of ShareNet’s improvement over the original methods calculated using the one-sided Wilcoxon signed-rank test. ShareNet obtains significant improvement over the original methods in all but one comparison (Pearson on mouse blood lineage)

We then used these reference networks to compare the accuracy of the cell type-specific networks estimated using the network inference algorithms alone to that of their corresponding networks estimated with the inclusion of ShareNet. For the two datasets and across the three network inference algorithms (six settings in total), we observed that the networks inferred using ShareNet frequently outperformed the baseline networks inferred in the absence of sharing (Fig. 3). The improvement of ShareNet was statistically significant in all of the settings (one-sided signed-rank test *p* < $10^{-5}$), with the sole exception of Pearson correlation on the mouse blood lineage dataset. However, we note that Pearson underperforms the other network inference algorithms without ShareNet on this dataset, registering AUPRC ratio values (performance relative to a random predictor) around 1 across the three reference networks, which likely limits the benefits of information sharing.

For the Tabula Muris dataset, networks inferred using Pearson correlation with ShareNet produced the highest AUPRC ratios, with particularly striking performance gains with respect to the STRING reference, especially given the high AUPRC ratios already attained with Pearson correlation alone. For the mouse blood lineage, the combination of GENIE3 and ShareNet yielded the highest AUPRC ratios. The networks inferred using this method demonstrated consistent improvements in accuracy when compared to the STRING network and non-specific ChIP-seq networks in addition to increased AUPRC ratios for a majority of the cell type-specific ChIP-seq networks.

#### 3.1.3 Holdout likelihood analysis

While quantifying agreement with reference networks provides a valuable means to assess the overall quality of predicted regulatory associations, this approach is understandably limited given the lack of comprehensive ground truth associations. As a complementary approach to evaluating the utility of ShareNet, we set out to compare the predictive power of learned networks with and without ShareNet on previously unseen samples. We follow the intuition that, if a model generalizes better to new data, the underlying regulatory associations in the model used for prediction are also more likely to be real associations. To this end, we leveraged BVS, an extended ShareNet model equipped with the ability to calculate the likelihood of observed gene expression levels (see Materials and methods). Note that the network inference methods considered in previous sections (Pearson correlation, GENIE3 and PIDC) cannot be used for this analysis due to their lack of a likelihood model.

**Fig. 4.**
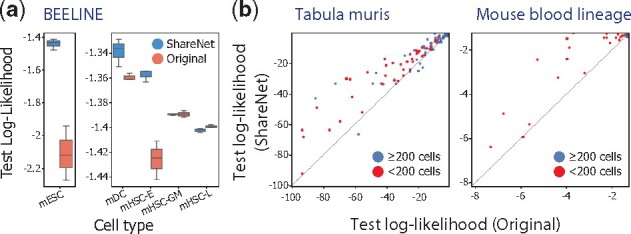
ShareNet improves holdout likelihood for (**a**) the BEELINE benchmark scRNA-seq dataset and (**b**) the Tabula Muris and mouse blood lineage scRNA-seq datasets. We compared the test log-likelihood for Bayesian variable selection (BVS) models trained with and without ShareNet. Each dot in a scatterplot (b) corresponds to a cell type in a dataset. Cell types represented by fewer than 200 cells were colored in red to mark undersampled cell types. ShareNet consistently led to higher likelihoods, most notably for cell types with limited sample sizes (mESC in (a) and red dots in (b))

For the BEELINE, Tabula Muris and mouse blood lineage datasets, we trained BVS with and without ShareNet, while holding out 20% of cells in each cell type for test likelihood calculation. We observed that the average test log likelihood values from models trained with ShareNet are consistently greater than or approximately equal to those from the model trained without it in the BEELINE dataset (Fig. 4a). Higher likelihoods indicate that the model is more effective at explaining gene expression patterns in the test data. The substantial improvement in average test log likelihood observed for mESC is noteworthy considering that mESC, at around 400 cells, is one of the most undersampled among the five cell types.

For the Tabula Muris and mouse blood lineage datasets, which feature more cell types and also a wider range of cell type population sizes, we also observed consistent improvement in the test likelihood across cell types for models trained with ShareNet compared to models trained without it (Fig. 4b). Notably, for cell types that feature fewer cells, the improvements in the test log likelihood values were the most pronounced. These results suggest that the information transferred across cell types by ShareNet includes meaningful biological patterns that help with the predictive modelling of gene expression.

We note that BVS may also be used to infer networks by ranking the edges according to the posterior inclusion probability $p(\gamma_{i,j}^{\left(c\right)}|e)$ of a regulator gene *i* in the predictive model for a target gene *j* in cell type *c* (Materials and methods). While BVS trained with and without ShareNet yielded only modest network accuracy results compared to Pearson, GENIE3 and PIDC across all our benchmark datasets, sharing information using ShareNet resulted in consistent accuracy improvements in the Tabula Muris and mouse blood lineage datasets (Supplementary Fig. S7).

### 3.2 ShareNet improves comparisons of cell type-specific regulatory associations across cell types and contexts

**Fig. 5.**
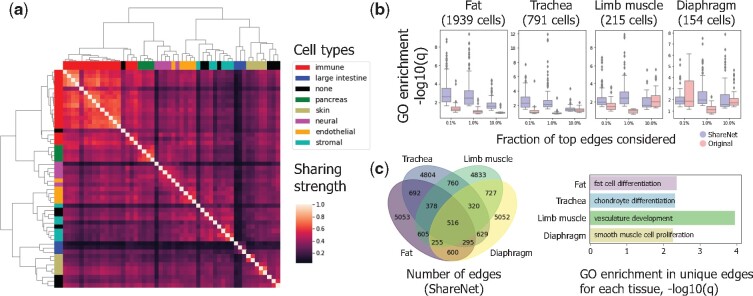
ShareNet learns meaningful sharing patterns across tissues and identifies regulatory associations specific to tissue-of-origin in the Tabula Muris dataset. (**a**) The learned cell type-cell type correlation matrix for the mixture component with the highest weight. Rows and columns are ordered by the output of hierarchical clustering and colored by the general cell type categories to which the 52 represented cell types belong. Learned sharing pattern is consistent with known cell type categories. (**b**) We compared the top 0.1%, 1% and 10% of edges in networks generated with and without ShareNet and identified edges uniquely associated with each. Enrichment *q* values were calculated for each biological process GO term using genes ranked by occurrence in the uniquely identified edges. Overall, only the edges unique to ShareNet display consistent GO enrichment. (**c**) We compared networks for MSCs isolated from different tissues and performed GO enrichment analysis on genes ranked by occurrence in the set of edges that were unique to each tissue-of-origin. Enriched GO terms are consistent with the corresponding tissues

ShareNet’s ability to identify cell type-specific regulatory associations with increased accuracy opens the door to a wide range of analyses exploring the unique wiring patterns that underlie cellular identity. We aimed to demonstrate this property on the Tabula Muris dataset, which consists of more than 100,000 cells from 20 organs and tissues (Schaum *et al*., 2018). From this vast collection of cells, we isolated all cell types that contained at least 100 cells, resulting in 52 cell types. We applied ShareNet on Pearson correlation networks, which achieved the best performance in our above analyses of this dataset. To understand what types of sharing patterns confer this improved accuracy, we examined the mixture components that are learned by our model and that define the manner in which cell types propagate information to one another. We identified the covariance matrix associated with the highest mixture weight in our model and clustered the cell types according to the corresponding correlation matrix. We observed that cell types that belong to the same broader cell type category based on annotations are clustered together (Fig. 5a), suggesting that the sharing patterns that are automatically learned by ShareNet capture biologically meaningful similarities among the cell types.

In addition to recapitulating known cell type categories, ShareNet enables us to perform more accurate comparisons of related cell types in different resident tissues. The Tabula Muris dataset includes mesenchymal stem cells (MSCs) isolated from the adipose tissue, trachea, limb muscle and diaphragm of mice. We sought to explore the specific differences in networks between these four types of MSCs with different tissues of origin. First, to assess the quality of MSCs networks produced by ShareNet, we examined the differences in the networks reconstructed with ShareNet and in its absence. Across a range of thresholds for retaining the top edges in each network, we identified the set of edges that are uniquely identified when using ShareNet relative to the baseline use case in its absence and vice versa. We then performed gene ontology (GO) enrichment analysis of genes ranked by their occurrence in these sets in an effort to characterize the signals either gained or removed as a result of information sharing (Eden *et al.*, 2009). Overall, we observed that the edges that are uniquely identified by ShareNet are consistently enriched for GO biological processes (Fig. 5b), including terms associated with differentiation and development (Supplementary Fig. S8). In contrast, the edges that are uniquely identified without ShareNet tend to have considerably less functional enrichment, implying that ShareNet omitted edges that are more likely to be spurious rather than functional cell type-specific interactions.

To compare the MSC networks produced by ShareNet across different tissues of origin, we took the top 1% of edges from each network and retained only the unique edges that were not included in the other MSC networks. GO enrichment analysis of these unique edges revealed significant enrichment for differentiation and development pathways that characterize the matching tissues from which each of the MSC populations were isolated (Fig. 5c). Notably, three of the four tissue-specific pathway enrichments (fat, limb muscle, diaphragm) could not be detected when using the networks inferred without ShareNet. These results provide further evidence that our method not only reduces the relevance of potential false positive edges in gene regulatory networks but also retains important regulatory associations that uniquely define the specific cell types that they represent.

### 3.3 ShareNet uncovers temporal regulatory changes during early mouse blood development

**Fig. 6.**
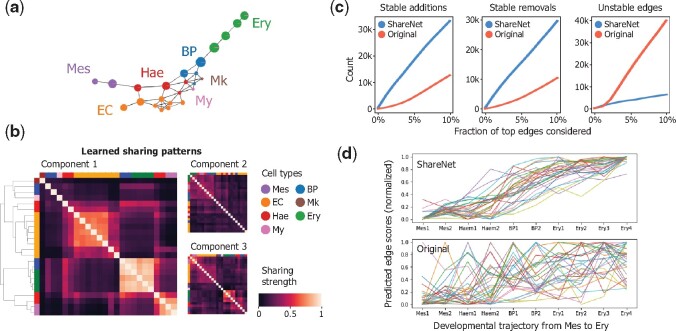
ShareNet enhances the detection of dynamic changes in regulatory associations along the early mouse blood differentiation trajectory. (**a**) Coarse graph abstraction of the mouse blood lineage produced by the PAGA algorithm (Wolf *et al.*, 2019). Nodes correspond to specific cell types and are colored by the general cell type categories. Mes: mesodermal cells, Ery: erythrocytes, Mk: megakaryocytes, Hae: haemato-endothelial progenitors, EC: endothelial cells, My: myeloid cells. (**b**) Cell type–cell type sharing patterns learned by ShareNet for the mouse blood lineage dataset. The correlation matrices of the mixture components with the highest mixture weights are depicted. Rows and columns are ordered by hierarchical clustering and colored by general cell type categories to which the 24 represented cell types belong. Learned sharing patterns are consistent with known cell type categories, and different components capture distinct sharing patterns. (**c**) The number of stably added, stably removed and unstable edges along the differentiation path from mesodermal to erythroid cells, for varying proportions of top edges included in the networks. ShareNet enhances the discovery of persistent shifts while suppressing unstable associations that are likely due to noise. (**d**) Temporal trajectories of inferred edge scores along the path from mesodermal to erythroid cells for ShareNet-GENIE3 (top) and GENIE3 without ShareNet (bottom). Displayed set of curves represent a random subset of stably added edges in the ShareNet output. Each trajectory is min-max normalized. ShareNet reveals persistent activation of regulatory associations that are obscured without ShareNet.

To demonstrate the utility of ShareNet for studying the rewiring of regulatory associations in dynamic biological processes, we leveraged it to reconstruct cell type-specific networks in the early mouse blood lineage. We used the aforementioned mouse blood lineage dataset, which contains erythroid (Ery), haemato-endothelial (Hae), blood progenitor (BP), endothelial (EC) and mixed mesodermal (Mes) cells collected from whole mouse embryos between the E6.5 and E8.5 stages of early embryonic development (Pijuan-Sala *et al.*, 2019) (Fig. 6a). We used GENIE3 with ShareNet to infer networks for these cell types, as this combination yielded the strongest performance for this dataset in our results above. A closer look at the sharing patterns automatically learned by ShareNet revealed that the captured relationships among cell types cohere with expert-annotated labels based on marker genes and their underlying trajectory (Fig. 6b). Furthermore, different mixture components captured distinct sharing patterns, which merit further exploration.

Using the inferred networks, we sought to investigate the regulatory rewiring patterns that define the part of the trajectory spanning the differentiation of Mes cells to Ery cells in the primitive wave of Ery cell formation (Pijuan-Sala *et al.*, 2019). To do so, we examined our inferred cell type-specific networks for the 10 cell types along the shortest path between Mes and Ery cells, based on the association graph constructed by the PAGA algorithm (Wolf *et al.*, 2019).

From this chain of networks, we aimed to identify regulatory interactions that are reliably activated or deactivated during the course of differentiation as a means of detecting persistent shifts in regulatory dynamics that confer cellular identity. We first searched for *stably added* edges in networks that are absent in the earliest Mes cells but emerge in an intermediate cell type along the trajectory, upon which they are stably maintained across the successive cell types until the terminal Ery cells. Across a range of percentage values that determine the proportion of putative edges to retain in each network, we found that the number of edges that meet this criterion is consistently higher in the networks inferred using ShareNet and GENIE3 compared to GENIE3 on its own (Fig. 6c). In addition, the underlying edge scores associated with this set of stably added edges demonstrated a clear increasing trend along the differentiation path, consistent with the fact that these edges are of increasing relevance to cells as they approach the erythroid lineage (Fig. 6d). On the contrary, the edge scores for the same set of edges in the GENIE3 networks do not show this trend and change erratically from cell type to cell type. In searching for edges that are *stably removed* across the differentiation trajectory, the set of networks inferred using ShareNet also presented notably more of these edges (Fig. 6c). Lastly, edges that are *unstable*, which we define to be those that change activity status more than four times over the course of the path of ten cell types, are more highly represented in the set of networks inferred in the absence of ShareNet. Given the limited plausibility of these unstable associations, we believe this observation reflects the higher noise levels in the underpowered GENIE3 networks; ShareNet is noticeably less affected by this potential artifact.

Furthermore, GO enrichment analysis on genes ranked by their occurrence in the set of stably added edges revealed enrichment for erythrocyte development (GO: 0048821, FDR $q=1.53\times 10^{-3}$), while a similar analysis performed on the GENIE3 networks without ShareNet revealed no such enrichment. In exploring the genes that are most implicated in these stably added edges, we found that *Tal1*, *Fam210b* and *Vgll4*, three genes are known to be implicated in erythroid differentiation, are among the top 10 most frequently occurring genes. *Tal1* is an essential transcription factor for erythropoiesis (Hall *et al.*, 2003; Wu *et al.*, 2014) and is involved in the activation and repression of a number of erythroid-specific target genes (Lausen *et al.*, 2010). Similarly, *Fam210b* and *Vgll4* are critical in regulating erythroid heme synthesis in terminal erythropoiesis (Wang *et al.*, 2020; Yien *et al.*, 2018). These results suggest that sharing information across cell types enhances our ability to detect meaningful patterns of network rewiring that are informative of the dynamics governing transitions in cellular identity.

### 3.4 Runtime and memory usage

The BEELINE, Tabula muris and mouse blood lineage datasets spanned a broad range of dataset sizes. The average total runtime for ShareNet was 2 min, 116 min and 411 min for the BEELINE, mouse blood lineage and Tabula muris datasets, respectively. Peak memory usage was approximately 664 MB, 11.9 GB and 111 GB for the same three datasets. All methods were run on a 2.40 GHz Intel Xeon E5-2695v2 central processing unit. A more in-depth discussion on the dataset factors affecting runtime and memory usage as well as future optimization strategies for reducing the computational burden can be found in Supplementary Materials.

## 4 Discussion and future work

The growing use of scRNA-seq data to characterize the diversity of cell types and cell states within populations of cells has led to the discovery of many new cell types. An important next step in comprehensively characterizing these cell types beyond their gene expression signatures is the elucidation of the underlying gene regulatory network structures that drive their unique expression profiles. In order to handle the issue of small samples sizes for cell types that are rare and underrepresented, we have presented ShareNet as a framework for automatically leveraging the overlap in network structures across similar cell types to improve estimations of cell type-specific networks. We have demonstrated that propagating information across cell types increases the accuracy of inferred networks in a range of datasets and experimental settings. As a result, we have been able to employ ShareNet to more finely characterize individual cell types from scRNA-seq datasets with respect to gene regulatory networks instead of just gene expression.

Our work drew inspiration from previous methods that leveraged joint inference of multiple related tasks to increase power for tasks that typically would be underpowered on their own. One domain in which such methods have been developed is statistical genetics. For example, Mash (Urbut *et al.*, 2019) is a powerful method for jointly estimating effect sizes for genetic associations across multiple conditions. Similar to our approach, Mash allows for a broad range of patterns of correlation among conditions, albeit restricted to a weighted average of pre-defined sharing patterns. For the task of inferring gene regulatory networks, several approaches have been developed for jointly estimating multiple networks, notably for bulk RNA-seq analysis, by linking networks from different species (Castro *et al.*, 2019; Koch *et al.*, 2017) or different points of time along a linear biological process (Ahmed and Xing, 2009; Song *et al.*, 2009). For each of these methods, though, the manner in which information is transferred between networks must be pre-specified and/or is highly restrictive. With single-cell datasets, the relationship between cell types is often unknown *a priori*, as many cell types are being detected for the first time. Furthermore, the diversity of cell types that appear in single-cell datasets presents a need to account for a wide range of possible sharing patterns. ShareNet addresses these idiosyncrasies of single-cell datasets, building upon the insights from the aforementioned related works.

Recent advancements in single-cell technologies have been made to simultaneously measure different biological aspects of individuals cells, including spatial gene expression, chromatin accessibility, and morphology (Zhu *et al.*, 2020). The integration of these multi-modal datasets (Singh *et al.*, 2020) in a manner similar to ShareNet would serve as an exciting next step for characterizing the intricate relationship between gene regulatory relationships and cellular identity in greater detail.

*Data Availability*: All data used in the study are publicly available. The BEELINE, Tabula muris, and mouse blood lineage datasets can be accessed at the following URLs, respectively: https://zenodo.org/record/3701939#.YJIHKy1h1bU, https://tabula-muris.ds.czbiohub.org, and https://github.com/MarioniLab/EmbryoTimecourse2018.

*Financial Support*: A.P.W. and B.B. are supported by NIH U01 CA250554 (to B.B.). H.C. is supported by Eric and Wendy Schmidt through the Schmidt Fellows Program at the Broad Institute. J.P. is supported by the NSF CAREER Award (1652815). 

*Conflict of Interest*: none declared.

## Supplementary Material

btab269_Supplementary_Data
