# Supplementary material for: Bayesian information sharing enhances detection of regulatory associations in rare cell types
Source: Bioinformatics. 2021 Jul 12;37(Suppl 1):i349–57. doi: 10.1093/bioinformatics/btab269 (PMC8275330; doi:10.1093/bioinformatics/btab269)
Supplement: btab269_Supplementary_Data [file btab269_supplementary_data.pdf]

Bayesian information sharing enhances detection of regulatory associations in rare cell types (Supplementary Materials)

1 Supplementary Figures

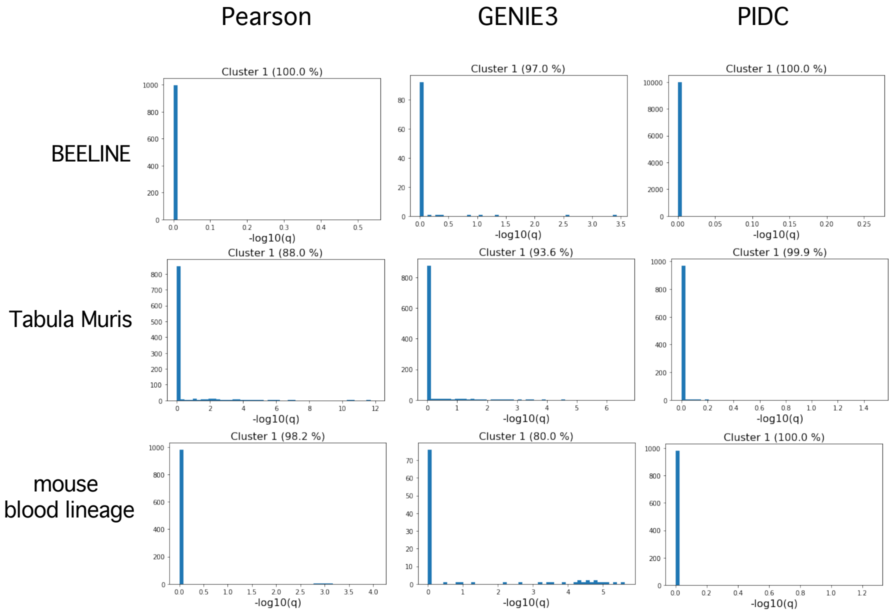

Supplementary Figure 1: Noise of network inference algorithms are approximately Gaussian. For a single cell type in each of the BEELINE, Tabula Muris, and mouse blood lineage datasets, we generated 50 sets of bootstrapped cell populations, each of which contain the same number of cells as the original population. We applied Pearson correlation, GENIE3, and PIDC on each of the bootstrapped cell populations and evaluated the distributions of 10,000 randomly selected edges across the 50 bootstrapped samples using the Shapiro-Wilk test of normality. The $-\log_{10}(q)$ values for the selected edges, calculated by applying Bonferonni correction on the test's p values, are depicted in the histograms. Percentages labeled on top of each histogram correspond to the proportion of edges that are associated with q values greater than 0.05.

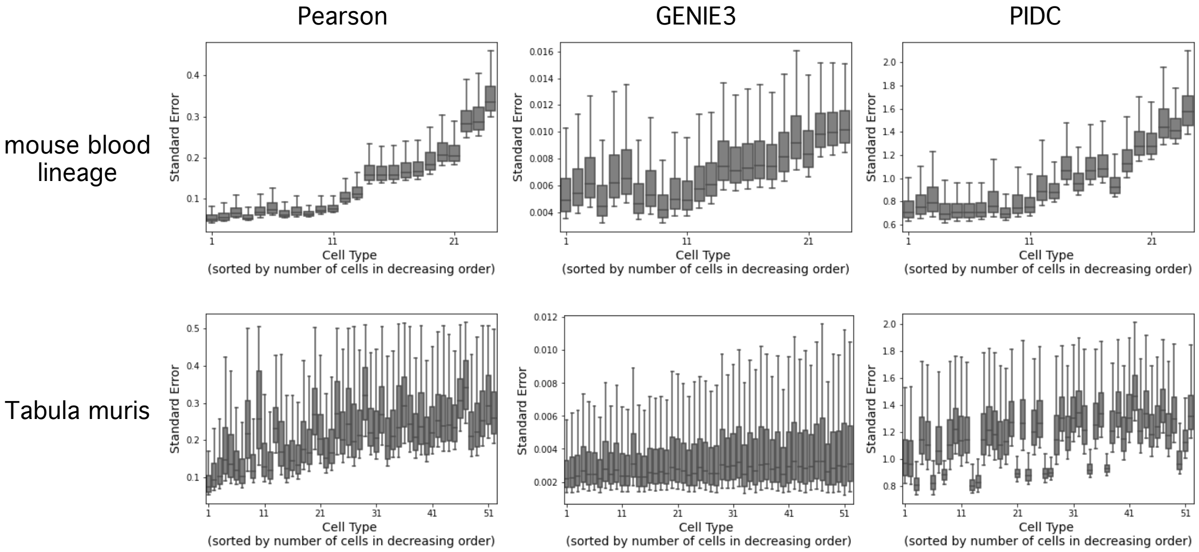

Supplementary Figure 2: Standard deviation estimates increase with decreasing sample size. For each cell type considered in the Tabula muris and mouse blood lineage datasets, we identified the top 10% of edges by estimated standard deviation based on five bootstrapped samples. Cell types are ordered according to the number of cells that represent them in the dataset. Cell type population sizes range from 1,939 cells to 103 cells in the Tabula Muris dataset, while the cell type population sizes for the mouse blood lineage dataset range from 1,860 cells to 30 cells.

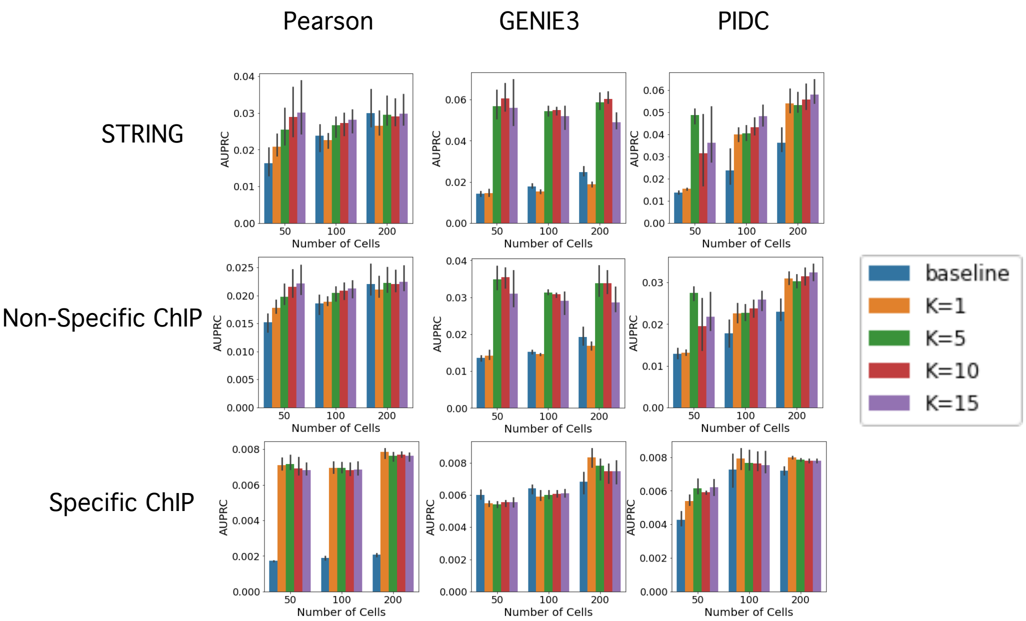

Supplementary Figure 3: Network accuracy results for ShareNet are robust with respect to the number of mixture components. We compared the accuracy of dendritic cell networks inferred with Pearson correlation, GENIE3, and PIDC, with and without ShareNet. The dendritic cell population is downsampled to 50, 100, and 200 cells. We tested a range of mixture component numbers for ShareNet across the set of downsampling conditions. The inferred dendritic cell networks were compared with the corresponding cell type-specific ChIP-seq networks. While increasing the number of mixture components appears to improve the accuracy of networks inferred with Pearson correlation for population sizes of 50, the effect of ShareNet on network accuracy is generally robust to the number of mixture components used, as long as multiple mixture components are used.

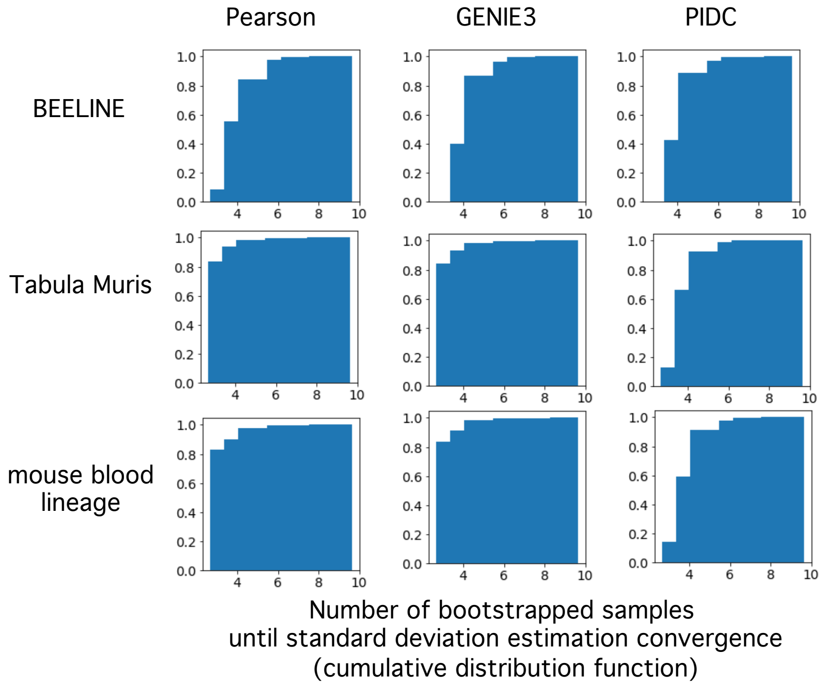

Supplementary Figure 4: Number of bootstrapped samples until convergence of edge score standard deviation estimates. For the a single cell type in each of the BEELINE, Tabula Muris, and mouse blood lineage datasets, we generated 50 sets of bootstrapped cell populations, each of which contain only 200 cells. We applied Pearson correlation, GENIE3, and PIDC on each of the bootstrapped cell populations and re-calculated the standard deviation of each edge's score upon the addition of each bootstrapped sample. We evaluated the number of bootstrapped samples required for the standard deviation estimate for each edge score to change by less than 20% and considered the distribution of the number of bootstrapped samples required to reach this convergence threshold. Around 5 bootstrapped samples were required to reach this convergence threshold for 80% of the edges across the various datasets and network inference algorithms.

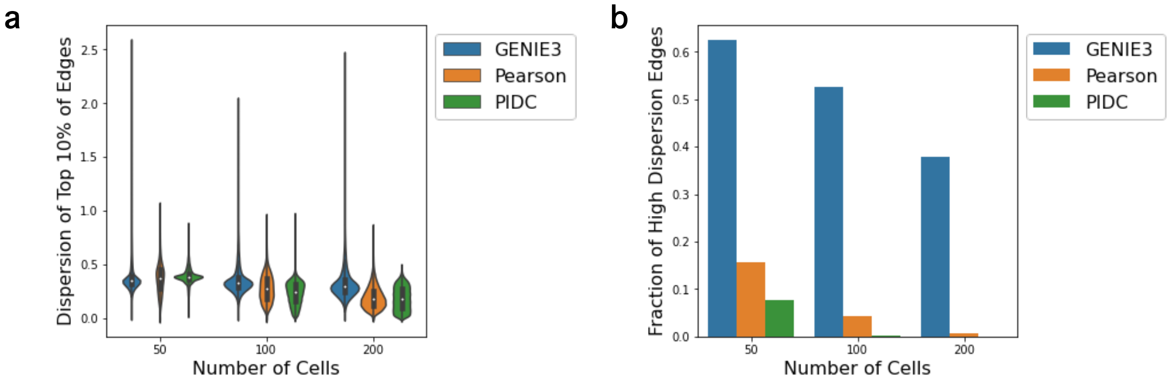

Supplementary Figure 5: GENIE3 edge scores are highly variable for small populations of cells in the BEELINE dataset. (a) The dispersion (standard deviation divided by mean) of the top 10% of edges ranked by edge score across networks inferred using GENIE3, Pearson correlation, and PIDC for cell population sizes of 50, 100, and 200 cells. (b) The fraction of edges associated with high dispersion (> 0.5) across the various network inference algorithms and cell population sizes.

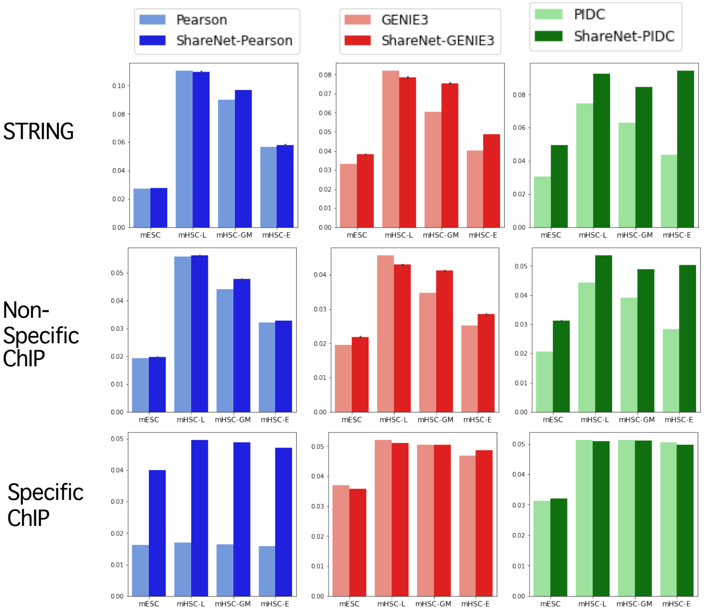

Supplementary Figure 6: ShareNet improves the accuracy of networks inferred in the presence of small populations of dendritic cells. We calculated AUPRC values for the four cell types that were included alongside the dendritic cells in the downsampling analyses. Networks inferred using ShareNet frequently outperformed the corresponding networks inferred without ShareNet, while also avoiding notable deterioration of accuracy despite sharing information with a downsampled cell type.

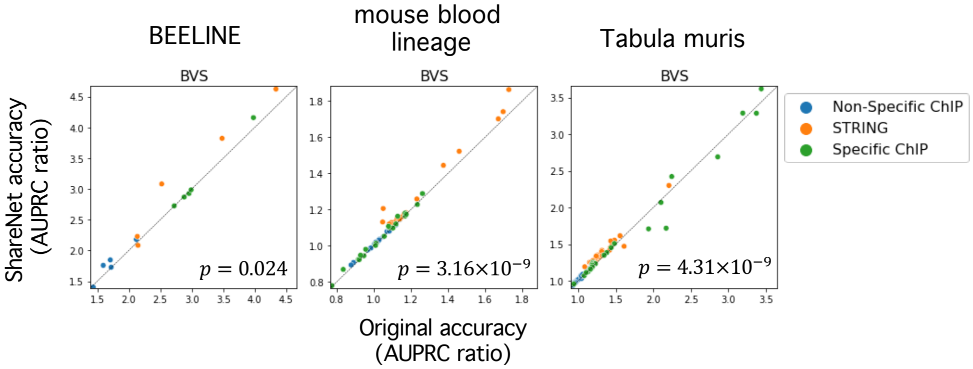

Supplementary Figure 7: ShareNet improves the accuracy of networks inferred using the Bayesian Variable Selection (BVS) model. Posterior probabilities of inclusion $p(\gamma_{i,j}^{(c)}|\boldsymbol{e})$ learned from BVS were used to construct networks for each cell type. p-values indicate the statistical significance of ShareNet's improvement over the original methods calculated using the one-sided Wilcoxon signed-rank test. ShareNet obtains significant improvement over the original methods in all but one comparison (BEELINE).

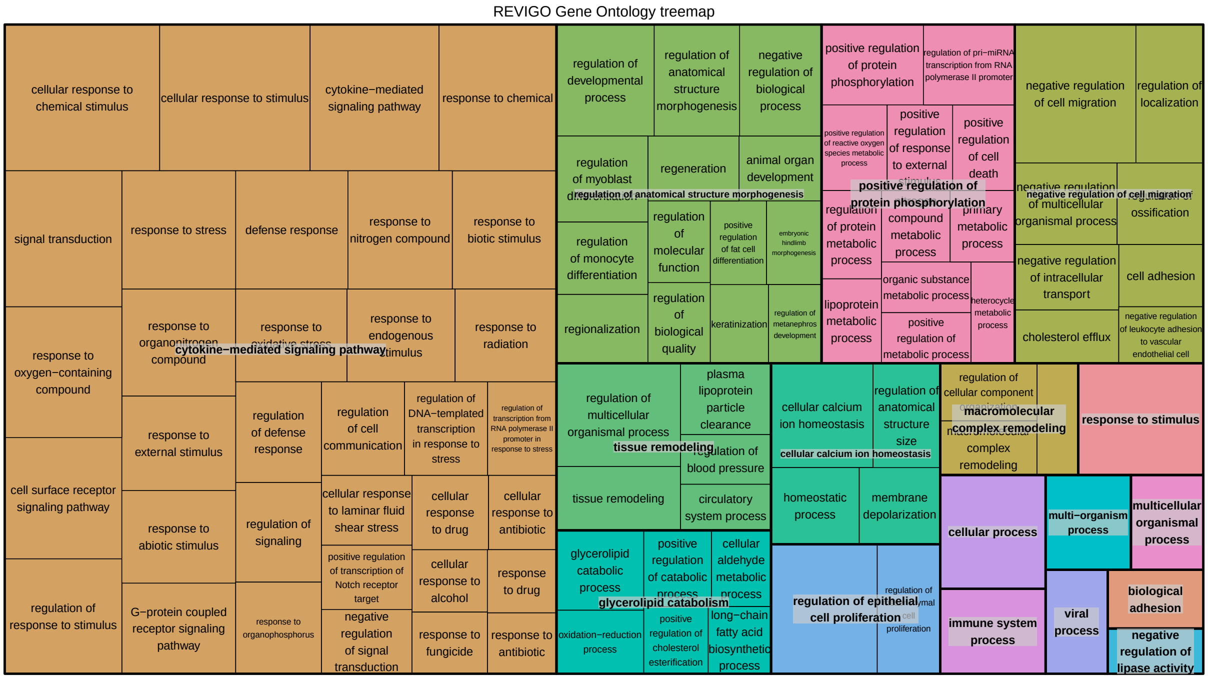

Supplementary Figure 8: Gene Ontology (GO) enrichment analysis on genes implicated in edges uniquely identified in ShareNet-Pearson networks relative to Pearson networks for MSCs in fat. Genes were ranked by frequency of occurrence in the uniquely identified edges when retaining the top 1% of edges in each network. The TreeMap plot was generated using REVIGO on the significant GO terms (FDR $q < 0.01$) identified in the enrichment analysis.

2 Derivation of ShareNet

In this section, we derive the parameter updates in the coordinate ascent variational inference procedure used in ShareNet.

2.1 Model Description

We first present the generative process for the Bayesian information sharing framework in full.

$$(\boldsymbol{\mu}_k, \boldsymbol{\Sigma}_k^{-1}) \sim \text{NW}(\mu_0, \beta_0, \Psi, \nu)$$
$$u_{i,j} \sim \text{Categorical}(1/K, ..., 1/K)$$
$$\boldsymbol{z}_{i,j}|u_{i,j} \sim \mathcal{N}(\boldsymbol{\mu}_{u_{i,j}}, \boldsymbol{\Sigma}_{u_{i,j}})$$
$$e_{i,j}^{(c)} \sim \mathcal{N}(z_{i,j}^{(c)}, \sigma_{i,j}^{(c)2})$$

Here, $e_{i,j}^{(c)}$ represents the observed score for the edge connecting gene i to gene j in cell type c, and we model it as a sample from a univariate Gaussian $\mathcal{N}(e_{i,j}^{(c)}|z_{i,j}^{(c)}, \sigma_{i,j}^{(c)2})$. The mean parameter $z_{i,j}^{(c)}$ of the Gaussian is a latent variable that represents the true score of the edge if calculated in an ideal scenario absent of noise. The variance parameter $\sigma_{i,j}^{(c)2}$ captures the degree of variation in the observed edge score. We treat this variance parameter $\sigma_{i,j}^{(c)2}$ as a hyperparameter, estimating it by bootstrap sampling each cell type in the dataset of interest, applying a chosen network inference algorithm on the bootstrapped samples, and then calculating the standard error, which we use as the standard deviation in this part of the model.

Working up the hierarchical model, the C-dimensional vector of latent variables $\boldsymbol{z}_{i,j} = (z_{i,j}^{(1)}, ..., z_{i,j}^{(C)})$ containing the true edge scores for edge (i,j) across the C cell types is a sample drawn from a mixture of multivariate Gaussian distributions with K mixture components. Each of the K Gaussians is parameterized by a mean $\boldsymbol{\mu}_k$ and an inverse covariance $\boldsymbol{\Sigma}_k^{-1}$ parameter that are drawn independently from the same Normal-Wishart prior. The set of K covariance matrices $\boldsymbol{\Sigma}_1, ..., \boldsymbol{\Sigma}_K$ represents the set of cell type-to-cell type sharing patterns.

Taking all of these distributions into consideration, the joint density for the full hierarchical model can be written as follows. For simplicity of representation, we denote each (regulator gene i-target gene j) combination with a unique subscript n.

$$p(\boldsymbol{\mu}, \boldsymbol{\Sigma}^{-1}, \boldsymbol{u}, \boldsymbol{z}, \boldsymbol{e}) = \prod_{k=1}^{K} p(\boldsymbol{\mu}_k, \boldsymbol{\Sigma}_k^{-1}) \prod_{n=1}^{N} \prod_{k=1}^{K} p(\boldsymbol{z}_n|\boldsymbol{\mu}_k, \boldsymbol{\Sigma}_k^{-1})^{u_{n,k}} \prod_{n=1}^{N} p(\boldsymbol{u}_n) p(\boldsymbol{e}_n|\boldsymbol{z}_n, \boldsymbol{D}_n^{-1})$$

Here, $\boldsymbol{D}_n = \text{diag}(1/\hat{\sigma}_n^{(1)2}, ..., 1/\hat{\sigma}_n^{(C)2})$ is the diagonal matrix containing the reciprocal of the squared standard error terms for edge n across cell types 1 to C.

2.2 Variational Inference

As the latent variable $\boldsymbol{z}$ represents the true edge scores, we are interested in calculating the posterior distribution of this latent variable given the observed edge scores.

$$p(\boldsymbol{z}|\boldsymbol{e}, \boldsymbol{\mu}, \boldsymbol{\Sigma}^{-1}, \boldsymbol{u}) = \frac{p(\boldsymbol{\mu}, \boldsymbol{\Sigma}^{-1}, \boldsymbol{u}, \boldsymbol{z}, \boldsymbol{e})}{p(\boldsymbol{e})}$$

We are similarly interested in calculating the posterior distributions of the remaining latent variables $(\boldsymbol{\mu}, \boldsymbol{\Sigma}^{-1}, \boldsymbol{u})$ given the observed edge scores. However, exact computation of these posterior distributions is intractable, as computing each of these distributions requires computing the evidence term $p(\boldsymbol{e})$ in the denominator.

$$p(\boldsymbol{e}) = \int\int\int\int \prod_{k=1}^{K} p(\boldsymbol{\mu}_k, \boldsymbol{\Sigma}_k^{-1}) \prod_{n=1}^{N} \prod_{k=1}^{K} p(\boldsymbol{z}_n|\boldsymbol{\mu}_k, \boldsymbol{\Sigma}_k^{-1})^{u_{n,k}} \prod_{n=1}^{N} p(\boldsymbol{u}_n) p(\boldsymbol{e}_n|\boldsymbol{z}_n, \boldsymbol{D}_n^{-1}) d\boldsymbol{\mu} d\boldsymbol{\Sigma}^{-1} d\boldsymbol{u} d\boldsymbol{z}$$

We can see that computing this evidence term requires integrating over $\boldsymbol{u}$, which is intractable to compute for even moderate sizes of N and K. As a result, we resort to variational inference as a tool for approximating these distributions.

2.2.1 Setup

In variational inference, we propose a family of approximating (variational) distributions and then seek to identify the member of this family of distributions that minimizes the KL divergence to the posterior distribution that we aim to approximate $p(\boldsymbol{U}|\boldsymbol{x})$.

$$q^*(\boldsymbol{U}) = \underset{q(\boldsymbol{U})}{\arg\min} \quad \text{KL}(q(\boldsymbol{U})||p(\boldsymbol{U}|\boldsymbol{x}))$$

Here, we define $\boldsymbol{U} = (\boldsymbol{\mu}, \boldsymbol{\Sigma}^{-1}, \boldsymbol{u}, \boldsymbol{z})$ to be the set of latent variables associated with our model and $\boldsymbol{x} = \boldsymbol{e}$ are the observed edge scores. Unfortunately, computation of the KL divergence objective function is also intractable because it requires computing $\log p(\boldsymbol{e})$.

$$\begin{aligned}
\text{KL}(q(\boldsymbol{\mu}, \boldsymbol{\Sigma}^{-1}, \boldsymbol{u}, \boldsymbol{z})||p(\boldsymbol{\mu}, \boldsymbol{\Sigma}^{-1}, \boldsymbol{u}, \boldsymbol{z}, \boldsymbol{e})) &= \mathbb{E}_q[\log q(\boldsymbol{\mu}, \boldsymbol{\Sigma}^{-1}, \boldsymbol{u}, \boldsymbol{z})] - \mathbb{E}_q[\log p(\boldsymbol{\mu}, \boldsymbol{\Sigma}^{-1}, \boldsymbol{u}, \boldsymbol{z}|\boldsymbol{e})] \\
&= \mathbb{E}_q[\log q(\boldsymbol{\mu}, \boldsymbol{\Sigma}^{-1}, \boldsymbol{u}, \boldsymbol{z})] - \mathbb{E}_q[\log p(\boldsymbol{\mu}, \boldsymbol{\Sigma}^{-1}, \boldsymbol{u}, \boldsymbol{z}, \boldsymbol{e})] + \log p(\boldsymbol{e})
\end{aligned}$$

We can, however, calculate an alternative objective, the ELBO (evidence lower bound), which is equivalent to the negative KL divergence up to an added constant.

$$\begin{aligned}
\text{ELBO}(q) &= \mathbb{E}_q[\log p(\boldsymbol{\mu}, \boldsymbol{\Sigma}^{-1}, \boldsymbol{u}, \boldsymbol{z}, \boldsymbol{e})] - \mathbb{E}_q[\log q(\boldsymbol{\mu}, \boldsymbol{\Sigma}^{-1}, \boldsymbol{u}, \boldsymbol{z})] \\
&= \mathbb{E}_q[\log p(\boldsymbol{e}|\boldsymbol{\mu}, \boldsymbol{\Sigma}^{-1}, \boldsymbol{u}, \boldsymbol{z})] - \text{KL}(q(\boldsymbol{\mu}, \boldsymbol{\Sigma}^{-1}, \boldsymbol{u}, \boldsymbol{z})||p(\boldsymbol{\mu}, \boldsymbol{\Sigma}^{-1}, \boldsymbol{u}, \boldsymbol{z}, \boldsymbol{e}))
\end{aligned}$$

We, therefore, seek to maximize the ELBO as our objective.

2.2.2 Variational family of distributions

In our setup, we assume that the variational distribution factorizes as

$$q(\boldsymbol{U}) = q(\boldsymbol{\mu}, \boldsymbol{\Sigma}^{-1}, \boldsymbol{u}, \boldsymbol{z}) = \prod_{k=1}^{K} q(\boldsymbol{\mu}_k, \boldsymbol{\Sigma}_k^{-1}) \prod_{n=1}^{N} q(\boldsymbol{z}_n) q(\boldsymbol{u}_n).$$

Given this family of distributions, our variational inference goal is to identify the member of this family that maximizes the ELBO.

2.2.3 Coordinate ascent variational inference: general

Let us first consider a general solution for an optimal factor in variational inference. For each factor in the variational distribution, its optimal distribution is proportional to the exponential of the expected log of the joint, where the expectation is taken with respect to the latent variables described by the remaining variational factors (18).

$$q_j^*(\boldsymbol{\theta}_j) \propto \exp\left(\mathbb{E}_{i \neq j}[\log p(\boldsymbol{\theta}, \boldsymbol{x})]\right)$$

Here, each $\boldsymbol{\theta}_j$ represents the set of latent variables that are being described by a variational factor j. We can apply this solution to determine the form of each of the factors in our variational distribution.

2.2.4 Coordinate ascent variational inference: $q(\boldsymbol{u}_n)$

Starting with the $q(\boldsymbol{u}_n)$ factor in our variational distribution, we first state the general form of the optimal solution.

$$q(\boldsymbol{u}_n) \propto \exp\left(\mathbb{E}_{q(\boldsymbol{\mu}, \boldsymbol{\Sigma}^{-1}, \boldsymbol{u}_{-n}, \boldsymbol{z}, \boldsymbol{e})}[\log p(\boldsymbol{\mu}, \boldsymbol{\Sigma}^{-1}, \boldsymbol{u}, \boldsymbol{z}, \boldsymbol{e})]\right)$$

Writing out this general solution, we have the following. Terms that do not depend on $\boldsymbol{u}_n$ are absorbed into a constant.

$$\begin{aligned}\log q^*(\boldsymbol{u}_n) &= \mathbb{E}_{q(\boldsymbol{\mu},\boldsymbol{\Sigma}^{-1},\boldsymbol{u}_{-n},\boldsymbol{z},\boldsymbol{e})}[\log p(\boldsymbol{\mu},\boldsymbol{\Sigma}^{-1},\boldsymbol{u},\boldsymbol{z},\boldsymbol{e})] + \text{constant} \\ &= \log p(\boldsymbol{u}_n) + \mathbb{E}_{q(\boldsymbol{\mu},\boldsymbol{\Sigma}^{-1},\boldsymbol{z})}\left[\sum_{k=1}^{K} u_{n,k}\log p(\boldsymbol{z}_n|\boldsymbol{\mu}_k,\boldsymbol{\Sigma}_k^{-1})\right] + \text{constant} \\ &= \sum_{n=1}^{N}\sum_{k=1}^{K} u_{n,k}\log\rho_{n,k} + \text{constant},\end{aligned}$$

where we define

$$\log\rho_{n,k} = \log\frac{1}{K} + \frac{1}{2}\mathbb{E}_{q(\boldsymbol{\Sigma}^{-1})}[\log|\boldsymbol{\Sigma}_k^{-1}|] - \frac{C}{2}\log(2\pi) - \frac{1}{2}\mathbb{E}_{q(\boldsymbol{\mu},\boldsymbol{\Sigma}^{-1},\boldsymbol{z})}\left[(\boldsymbol{z}_n-\boldsymbol{\mu}_k)^T\boldsymbol{\Sigma}_k^{-1}(\boldsymbol{z}_n-\boldsymbol{\mu}_k)\right]. \quad (1)$$

If we exponentiate both sides, our optimal solution takes on the form $q(\boldsymbol{u}_n) \propto \prod_{k=1}^{K}\rho_{n,k}^{u_{n,k}}$, which when normalized is a categorical distribution.

$$q(\boldsymbol{u}) \propto \prod_{n=1}^{N}\prod_{k=1}^{K}\tilde{\phi}_{n,k}^{u_{n,k}}, \qquad \text{where} \quad \tilde{\phi}_{n,k} = \frac{\rho_{n,k}}{\sum_{j=1}^{K}\rho_{n,j}}.$$

From this result, we also have $\mathbb{E}_{q(\boldsymbol{u})}[u_{n,k}] = \tilde{\phi}_{n,k}$, which will be used in our derivations of the other variational factors below. We will also return to the expectation terms within Equation 1 in the derivations below.

2.2.5 Coordinate ascent variational inference: $q(\boldsymbol{\mu}_k, \boldsymbol{\Sigma}_k^{-1})$

We apply a similar procedure for determining the optimal solution for the $q(\boldsymbol{\mu}_k, \boldsymbol{\Sigma}_k^{-1})$ factor. The general solution for this factor is as follows.

$$\begin{aligned}\log q^*(\boldsymbol{\mu}_k,\boldsymbol{\Sigma}_k^{-1}) &= \mathbb{E}_{\boldsymbol{\mu}_{-k},\boldsymbol{\Sigma}_{-k}^{-1},\boldsymbol{z},\boldsymbol{u},\boldsymbol{e}}[\log p(\boldsymbol{\mu},\boldsymbol{\Sigma}^{-1},\boldsymbol{u},\boldsymbol{z},\boldsymbol{e})] + \text{constant} \\ &= \log p(\boldsymbol{\mu}_k,\boldsymbol{\Sigma}_k^{-1}) + \mathbb{E}_{q(\boldsymbol{u},\boldsymbol{z})}\left[\sum_{n=1}^{N} u_{n,k}\log p(\boldsymbol{z}_n|\boldsymbol{\mu}_k,\boldsymbol{\Sigma}_k^{-1})\right] + \text{constant} \\ &= \log p(\boldsymbol{\mu}_k,\boldsymbol{\Sigma}_k^{-1}) + \sum_{n=1}^{N}\mathbb{E}_{q(\boldsymbol{u})}[u_{n,k}]\mathbb{E}_{q(\boldsymbol{z})}\left[\log p(\boldsymbol{z}_n|\boldsymbol{\mu}_k,\boldsymbol{\Sigma}_k^{-1})\right] + \text{constant} \\ &= \log p(\boldsymbol{\mu}_k,\boldsymbol{\Sigma}_k^{-1}) + \sum_{n=1}^{N}\tilde{\phi}_{n,k}\mathbb{E}_{q(\boldsymbol{z})}\left[\log p(\boldsymbol{z}_n|\boldsymbol{\mu}_k,\boldsymbol{\Sigma}_k^{-1})\right] + \text{constant}\end{aligned}$$

Because our prior over $(\boldsymbol{\mu}_k, \boldsymbol{\Sigma}_k^{-1})$ is a Normal-Wishart distribution and the distribution over each z_n in our hierarchical model is Gaussian, our optimal solution is a Normal-Wishart distribution parameterized as follows.

$$\begin{aligned}q(\boldsymbol{\mu}_k,\boldsymbol{\Sigma}_k^{-1}) &= \mathrm{NW}(\boldsymbol{\mu}_k,\boldsymbol{\Sigma}_k^{-1}|\tilde{\boldsymbol{a}}_k,\tilde{\beta}_0,\tilde{\boldsymbol{B}}_k,\tilde{\nu}_k) \\ \tilde{\boldsymbol{a}}_k &= \frac{\beta_0\mu_0 + \sum_{n=1}^{N}\tilde{\phi}_{n,k}\mathbb{E}_q[\boldsymbol{z}_n]}{\beta_0 + N_k} \\ \tilde{\boldsymbol{B}}_k^{-1} &= \frac{(\mu_0 - \sum_{n=1}^{N}\tilde{\phi}_{n,k}\mathbb{E}_{q(\boldsymbol{z})}[\boldsymbol{z}_n]/N_k)(\mu_0 - \sum_{n=1}^{N}\tilde{\phi}_{n,k}\mathbb{E}_{q(\boldsymbol{z})}[\boldsymbol{z}_n]/N_k)^T}{1/\beta_0 + 1/N_k} \\ &\quad + \boldsymbol{\Psi}^{-1} + \sum_{n=1}^{N}\tilde{\phi}_{n,k}\mathbb{E}_{q(\boldsymbol{z})}\left[(\boldsymbol{z}_n-\boldsymbol{\mu}_k)(\boldsymbol{z}_n-\boldsymbol{\mu}_k)^T\right] \\ N_k &= \sum_{n=1}^{N}\tilde{\phi}_{n,k}\end{aligned}$$

$$\tilde{\nu}_k = \nu + \sum_{n=1}^{N} \tilde{\phi}_{n,k}$$

Similar to the above, we will return to the expectation terms expressed in these equations in a subsequent section.

2.2.6 Coordinate ascent variational inference: $q(\boldsymbol{z}_n)$

$$\begin{aligned}
\log q^*(\boldsymbol{z}_n) &= \sum_{k=1}^{K} \mathbb{E}_{\boldsymbol{\mu}, \boldsymbol{\Sigma}^{-1}, \boldsymbol{u}}[u_{n,k} \log p(\boldsymbol{z}_n | \boldsymbol{\mu}_k, \boldsymbol{\Sigma}_k^{-1})] + \log p(\boldsymbol{e}_n | \boldsymbol{z}_n, \boldsymbol{D}_n^{-1}) + \text{constant} \\
&= \sum_{k=1}^{K} \mathbb{E}_{\boldsymbol{\mu}, \boldsymbol{\Sigma}^{-1}, \boldsymbol{u}}[u_{n,k} \log \mathcal{N}(\boldsymbol{z_n} | \boldsymbol{\mu}, \boldsymbol{\Sigma}^{-1})] + \log \mathcal{N}(\boldsymbol{e}_n | \boldsymbol{z}_n, \boldsymbol{D}_n^{-1}) + \text{constant}
\end{aligned}$$

Because the prior over $\boldsymbol{z}_n$ in the hierarchical model is Gaussian and the distribution over $\boldsymbol{z}_n$ is also Gaussian, $q^*(\boldsymbol{z}_n)$ is takes on the following Gaussian distribution.

$$\begin{aligned}
q^*(\boldsymbol{z}_n) &= \mathcal{N}(\tilde{\boldsymbol{m}}_n, \tilde{\boldsymbol{S}}_n) \\
\tilde{\boldsymbol{S}}_n &= \left(\sum_{k=1}^{K} \tilde{\phi}_{n,k} (\tilde{\nu}_k \tilde{\boldsymbol{B}}_k) + \boldsymbol{D}_n \right)^{-1} \\
\tilde{\boldsymbol{m}}_n &= \tilde{\boldsymbol{S}}_n \left(\sum_{k=1}^{K} \tilde{\phi}_{n,k} \tilde{\boldsymbol{\mu}}_k (\tilde{\nu}_k \tilde{\boldsymbol{B}}_k) + \boldsymbol{e}_n \boldsymbol{D}_n \right)
\end{aligned}$$

2.2.7 Full set of coordinate ascent updates

Using the above expressions for the various factors in our variational distribution, we can write out the complete set of corresponding variational parameter update equations.

For the variational factor $q(\boldsymbol{\mu}_k, \boldsymbol{\Sigma}_k^{-1})$, the update equations for $\tilde{\boldsymbol{a}}_k$ and $\tilde{\boldsymbol{B}}_k^{-1}$ are as follows.

$$\begin{aligned}
\tilde{\boldsymbol{a}}_k &= \frac{\beta_0 \mu_0 + \sum_{n=1}^{N} \tilde{\phi}_{n,k} \mathbb{E}_q[\boldsymbol{z}_n]}{\beta_0 + N_k} = \frac{\beta_0 \mu_0 + \sum_{n=1}^{N} \tilde{\phi}_{n,k} \tilde{\boldsymbol{m}}_n}{\beta_0 + N_k} \\
\tilde{\boldsymbol{B}}_k^{-1} &= \frac{(\mu_0 - \sum_{n=1}^{N} \tilde{\phi}_{n,k} \mathbb{E}_{q(\boldsymbol{z})}[\boldsymbol{z}_n]/N_k)(\mu_0 - \sum_{n=1}^{N} \tilde{\phi}_{n,k} \mathbb{E}_{q(\boldsymbol{z})}[\boldsymbol{z}_n]/N_k)^T}{1/\beta_0 + 1/N_k} \\
&\quad + \boldsymbol{\Psi}^{-1} + \sum_{n=1}^{N} \tilde{\phi}_{n,k} \mathbb{E}_{q(\boldsymbol{z})} \Big[(\boldsymbol{z}_n - \boldsymbol{\mu}_k)(\boldsymbol{z}_n - \boldsymbol{\mu}_k)^T \Big] \\
&= \frac{(\mu_0 - \sum_{n=1}^{N} \tilde{\phi}_{n,k} \tilde{\boldsymbol{m}}_n / N_k)(\mu_0 - \sum_{n=1}^{N} \tilde{\phi}_{n,k} \tilde{\boldsymbol{m}}_n / N_k)^T}{1/\beta_0 + 1/N_k} \\
&\quad + \boldsymbol{\Psi}^{-1} + \sum_{n=1}^{N} \tilde{\phi}_{n,k} \Big((\tilde{\boldsymbol{m}}_n - \tilde{\boldsymbol{\mu}}_k)(\tilde{\boldsymbol{m}}_n - \tilde{\boldsymbol{\mu}}_k)^T + \tilde{\boldsymbol{S}}_n \Big)
\end{aligned}$$

For the $q(\boldsymbol{u}_n)$ factor, we had Equation 1, which contained the following expectation terms that we can now write out.

$$\mathbb{E}_{q(\boldsymbol{\Sigma}^{-1})}[\log |\boldsymbol{\Sigma}_k^{-1}|] = \sum_{c=1}^{C} \psi \left(\frac{\tilde{\nu}_k + 1 - c}{2} \right) + C \log 2 + \log |\tilde{\boldsymbol{B}}_k|$$

$$\mathbb{E}_{q(\boldsymbol{\mu}, \boldsymbol{\Sigma}^{-1}, \boldsymbol{z})} \Big[(\boldsymbol{z}_n - \boldsymbol{\mu}_k)^T \boldsymbol{\Sigma}_k^{-1} (\boldsymbol{z}_n - \boldsymbol{\mu}_k) \Big] = (\tilde{\boldsymbol{m}}_n - \tilde{\boldsymbol{\mu}}_k)^T (\tilde{\nu}_k \tilde{\boldsymbol{B}}_k)(\tilde{\boldsymbol{m}}_n - \tilde{\boldsymbol{\mu}}_k) + \text{Tr}((\tilde{\nu}_k \tilde{\boldsymbol{B}}_k) \tilde{\boldsymbol{S}}_n) + \mathbb{E}_{q(\boldsymbol{\Sigma}^{-1})}[\text{Tr}(\tilde{\beta}_k^{-1} \boldsymbol{I})]$$

$$= (\tilde{\boldsymbol{m}}_n - \tilde{\boldsymbol{\mu}}_k)^T(\tilde{\nu}_k\tilde{\boldsymbol{B}}_k)(\tilde{\boldsymbol{m}}_n - \tilde{\boldsymbol{\mu}}_k) + \mathrm{Tr}((\tilde{\nu}_k\tilde{\boldsymbol{B}}_k)\tilde{\boldsymbol{S}}_n) + C\tilde{\beta}_k^{-1}$$

Using the above two equations, our revised expression for Equation 1 then becomes

$$\begin{aligned}\log\rho_{n,k} =& \log\frac{1}{K} + \frac{1}{2}\left(\sum_{c=1}^{C}\psi\left(\frac{\tilde{\nu}_k + 1 - c}{2}\right) + C\log 2 + \log|\tilde{\boldsymbol{B}}_k|\right) - \frac{C}{2}\log(2\pi)\\ &- \frac{1}{2}\left((\tilde{\boldsymbol{m}}_n - \tilde{\boldsymbol{\mu}}_k)^T(\tilde{\nu}_k\tilde{\boldsymbol{B}}_k)(\tilde{\boldsymbol{m}}_n - \tilde{\boldsymbol{\mu}}_k) + \mathrm{Tr}((\tilde{\nu}_k\tilde{\boldsymbol{B}}_k)\tilde{\boldsymbol{S}}_n) + C\tilde{\beta}_k^{-1}\right)\end{aligned}$$

We now have a complete set of coordinate ascent update equations for our variational parameters.

$$\tilde{\boldsymbol{a}}_k = \frac{\beta_0\mu_0 + \sum_{n=1}^{N}\tilde{\phi}_{n,k}\tilde{\boldsymbol{m}}_n}{\beta_0 + N_k} \tag{2}$$

$$\begin{aligned}\tilde{\boldsymbol{B}}_k^{-1} =& \frac{(\mu_0 - \sum_{n=1}^{N}\tilde{\phi}_{n,k}\tilde{\boldsymbol{m}}_n/N_k)(\mu_0 - \sum_{n=1}^{N}\tilde{\phi}_{n,k}\tilde{\boldsymbol{m}}_n/N_k)^T}{1/\beta_0 + 1/N_k}\\ &+ \boldsymbol{\Psi}^{-1} + \sum_{n=1}^{N}\tilde{\phi}_{n,k}\left((\tilde{\boldsymbol{m}}_n - \tilde{\boldsymbol{\mu}}_k)(\tilde{\boldsymbol{m}}_n - \tilde{\boldsymbol{\mu}}_k)^T + \tilde{\boldsymbol{S}}_n\right)\end{aligned} \tag{3}$$

$$N_k = \sum_{n=1}^{N}\tilde{\phi}_{n,k} \tag{4}$$

$$\tilde{\nu}_k = \nu + \sum_{n=1}^{N}\tilde{\phi}_{n,k} \tag{5}$$

$$\tilde{\phi}_{n,k} = \frac{\rho_{n,k}}{\sum_{j=1}^{K}\rho_{n,j}} \tag{6}$$

$$\begin{aligned}\log\rho_{n,k} =& \log\frac{1}{K} + \frac{1}{2}\left(\sum_{c=1}^{C}\psi\left(\frac{\tilde{\nu}_k + 1 - c}{2}\right) + C\log 2 + \log|\tilde{\boldsymbol{B}}_k|\right) - \frac{C}{2}\log(2\pi)\\ &- \frac{1}{2}\left((\tilde{\boldsymbol{m}}_n - \tilde{\boldsymbol{\mu}}_k)^T(\tilde{\nu}_k\tilde{\boldsymbol{B}}_k)(\tilde{\boldsymbol{m}}_n - \tilde{\boldsymbol{\mu}}_k) + \mathrm{Tr}((\tilde{\nu}_k\tilde{\boldsymbol{B}}_k)\tilde{\boldsymbol{S}}_n) + C\tilde{\beta}_k^{-1}\right)\end{aligned} \tag{7}$$

$$\tilde{\boldsymbol{S}}_n = \left(\sum_{k=1}^{K}\tilde{\phi}_{n,k}(\tilde{\nu}_k\tilde{\boldsymbol{B}}_k) + \boldsymbol{D}_n\right)^{-1} \tag{8}$$

$$\tilde{\boldsymbol{m}}_n = \tilde{\boldsymbol{S}}_n\left(\sum_{k=1}^{K}\tilde{\phi}_{n,k}\tilde{\boldsymbol{\mu}}_k(\tilde{\nu}_k\tilde{\boldsymbol{B}}_k) + \boldsymbol{e}_n\boldsymbol{D}_n\right) \tag{9}$$

3 Derivation of Bayesian Variable Selection (BVS)

3.1 Model Description

Our derivation of the Bayesian variable selection with ShareNet as prior contains many of the same terms as above, as we seek to leverage a mixture of Gaussians to share information across cell types. As a result, the distributions that are used in this model overlap to some degree with the ones described earlier. In the Bayesian variable selection with ShareNet model, the generative process is as follows.

$$(\boldsymbol{\mu}_k, \boldsymbol{\Sigma}_k^{-1}) \sim \text{NW}(\mu_0, \beta_0, \Psi, \nu)$$
$$u_{i,j} \sim \text{Categorical}(1/K, ..., 1/K)$$
$$\boldsymbol{z}_{i,j}|u_{i,j} \sim \mathcal{N}(\boldsymbol{\mu}_{u_{i,j}}, \boldsymbol{\Sigma}_{u_{i,j}})$$

$$\gamma_{i,j}^{(c)} \sim \text{Bernoulli}(g(z_{i,j}^{(c)}))$$
$$\beta_{i,j}^{(c)}|\gamma_{i,j}^{(c)} \sim \begin{cases} \mathcal{N}(0, \sigma_\beta^2) & \text{if } \gamma_{i,j}^{(c)} = 1, \\ \delta_0(\beta_{i,j}^{(c)}) & \text{otherwise,} \end{cases}$$
$$y_{j,n}^{(c)} \sim \mathcal{N}(\sum_i \boldsymbol{X}_{i,n}^{(c)} \beta_{i,j}^{(c)} \gamma_{i,j}^{(c)}, \sigma_\epsilon^2)$$

Here, $\beta_{i,j}^{(c)}$ represents the coefficient of the linear model describing effect of regulator gene i on target gene j, and $\gamma_{i,j}^{(c)}$ is a corresponding binary indicator variable that indicates whether or not regulator gene i is to be included in the network model for cell type c. We also include "spike and slab" priors (17) on $\beta_{i,j}^{(c)}$ and $\gamma_{i,j}^{(c)}$.

Based on our model, we have the following joint density for our Bayesian variable selection problem.

$$\begin{aligned}
\log p(\boldsymbol{\mu}, \boldsymbol{\Sigma}^{-1}, \boldsymbol{u}, \boldsymbol{y}, \boldsymbol{\gamma}, \boldsymbol{\beta}, \boldsymbol{z}|\boldsymbol{X}) = \sum_j \sum_{c=1}^{C} \Bigg\{ &-\frac{N^{(c)}}{2}\log(2\pi\sigma_\epsilon) - \frac{\boldsymbol{y}_j^{(c)T}\boldsymbol{y}_j^{(c)}}{2\sigma_{\epsilon^{(c)}}^2} + \frac{1}{\sigma_{\epsilon^{(c)}}^2} \sum_{i \in R(j)} \gamma_{j,i}^{(c)} \beta_{j,i}^{(c)} \boldsymbol{X}_i^{(c)T} \boldsymbol{y}_j^{(c)} \\
&- \frac{1}{2\sigma_{\epsilon^{(c)}}^2} \sum_{i \in R(j)} \sum_{i' \neq i} (\gamma_{j,i'}^{(c)} \beta_{j,i'}^{(c)})(\gamma_{j,i}^{(c)} \beta_{j,i}^{(c)}) \boldsymbol{X}_{i'}^{(c)T} \boldsymbol{X}_i^{(c)} \\
&+ \sum_j -\frac{1}{2\sigma_{\epsilon^{(c)}}^2} \sum_{i \in R(j)} (\gamma_{j,i}^{(c)} \beta_{j,i}^{(c)})^2 \boldsymbol{X}_i^{(c)T} \boldsymbol{X}_i^{(c)} \Bigg\} \\
&+ \sum_j \sum_{c=1}^{C} \sum_{i \in R(j)} \left(\frac{-\log(2\pi\sigma_\beta^2)}{2} - \frac{\beta_{j,i}^{(c)2}}{2\sigma_\beta^2} \right) \\
&+ \sum_j \sum_{c=1}^{C} \sum_{i \in R(j)} \gamma_{j,i}^{(c)} \log(g(z_{j,i}^{(c)})) + (1 - \gamma_{j,i}^{(c)}) \log(1 - g(z_{j,i}^{(c)})) \\
&+ \sum_j \sum_{k=1}^{K} \sum_{n=1}^{N} u_{(j,i),k} \left(-\frac{C}{2}\log(2\pi) + \frac{1}{2}\log|\boldsymbol{\Sigma}_k^{-1}| - \frac{1}{2}(\boldsymbol{z}_{j,i} - \boldsymbol{\mu}_k)^T \boldsymbol{\Sigma}_k^{-1} (\boldsymbol{z}_{j,i} - \boldsymbol{\mu_k}) \right) \\
&+ \sum_{k=1}^{K} -\frac{C}{2}\log(2\pi) - \frac{1}{2}\log|(\beta_0 \boldsymbol{\Psi})^{-1}| - \frac{1}{2}(\boldsymbol{\mu} - \boldsymbol{\mu}_0)^T (\beta_0 \boldsymbol{\Psi})(\boldsymbol{\mu_k} - \boldsymbol{m}_0) \\
&+ \sum_{k=1}^{K} \frac{\nu - C - 1}{2} \log|\boldsymbol{\Sigma}_k^{-1}| - \frac{1}{2}\text{Tr}(\boldsymbol{\Psi}^{-1}\boldsymbol{\Sigma}_k^{-1}) - \log\left(\left(\frac{\nu}{2\pi}\right)^{C/2} |\boldsymbol{\Psi}|^{\nu/2} 2^{C\nu/2} \Gamma_C(\nu/2) \right)
\end{aligned}$$

3.2 Variational Inference

Similar to the previous model, we seek to define a family of variational distributions from which we identify an optimal approximating distribution. In this scenario, the variational distribution factorizes as follows.

$$\begin{aligned} q(\boldsymbol{\gamma}_j, \boldsymbol{\beta}_j, \boldsymbol{z}_j, \boldsymbol{S}^{-1}) &= \Big(\prod_{c=1}^{C}\prod_{i\in R(j)} q(\beta_{j,i}^{(c)}, \gamma_{j,i}^{(c)})\Big)\Big(\prod_{i\in R(j)} q(\boldsymbol{z}_{j,i})\Big)\Big(q(\boldsymbol{S}^{-1})\Big) \\ &= \Big(\prod_{c=1}^{C}\prod_{i\in R(j)} q(\beta_{j,i}^{(c)}|\gamma_{j,i}^{(c)})q(\gamma_{j,i}^{(c)})\Big)\Big(\prod_{i\in R(j)} q(\boldsymbol{z}_{j,i})\Big)\Big(q(\boldsymbol{S}^{-1})\Big) \end{aligned}$$

We also define the distributions for the following set of variational factors.

$$\begin{aligned} q(\beta_{j,i}^{(c)}|\gamma_{j,i}^{(c)}) &= \begin{cases} \mathcal{N}(\beta_{j,i}^{(c)}|\mu_{j,i}^{(c)}, s_{j,i}^{(c)2}) & \text{if } \gamma_{j,i}^{(c)} = 1 \\ \delta_0(\beta_{j,i}^{(c)}) & \text{otherwise} \end{cases} \\ q(\gamma_{j,i}^{(c)}) &= (\alpha_{j,i}^{(c)})^{\gamma_{j,i}^{(c)}}(1-\alpha_{j,i}^{(c)})^{1-\gamma_{j,i}^{(c)}} \\ q(\boldsymbol{z}_{j,i}) &= \mathcal{N}(\boldsymbol{z}_{j,i}|\tilde{\boldsymbol{m}}_{j,i}, \tilde{\boldsymbol{S}}_{j,i}) \end{aligned}$$

3.2.1 ELBO

Given our joint density and the general form of our variational distributions, we can write out the ELBO.

$$\begin{aligned} \text{ELBO}(q) = \mathcal{L}(q) &= \mathbb{E}_{q(\boldsymbol{\gamma}_j,\boldsymbol{\beta}_j,\boldsymbol{z}_j,\boldsymbol{S}^{-1})}[\log P(\boldsymbol{X}_j, \boldsymbol{\gamma}_j, \boldsymbol{\beta}_j|\boldsymbol{X}_{-j}, \boldsymbol{z}_j, \boldsymbol{S}^{-1}, \theta) - \log q(\boldsymbol{\gamma}_j, \boldsymbol{\beta}_j, \boldsymbol{z}_j, \boldsymbol{S}^{-1})] \\ &= \mathbb{E}_{q(\boldsymbol{\gamma}_j,\boldsymbol{\beta}_j,\boldsymbol{z}_j,\boldsymbol{S}^{-1})}[\log P(\boldsymbol{X}_j, \boldsymbol{\gamma}_j, \boldsymbol{\beta}_j, \boldsymbol{z}_j, \boldsymbol{S}^{-1}|\boldsymbol{X}_{-j}, \theta)] - \mathbb{E}_{q(\boldsymbol{\gamma}_j,\boldsymbol{\beta}_j,\boldsymbol{z}_j,\boldsymbol{S}^{-1})}[\log q(\boldsymbol{\gamma}_j, \boldsymbol{\beta}_j, \boldsymbol{z}_j, \boldsymbol{S}^{-1})] \end{aligned}$$

We split up the first and second terms in the ELBO and consider them separately at first for simplicity.

$$\begin{aligned} \mathbb{E}_q\Big[\log p(\boldsymbol{y}_j, \boldsymbol{\gamma}_j, \boldsymbol{\beta}_j, \boldsymbol{z}_j, \boldsymbol{S}^{-1}|\boldsymbol{X}_j)\Big] = \sum_{c=1}^{C}\Big\{ &-\frac{N^{(c)}}{2}\log(2\pi\sigma_\epsilon) - \frac{\boldsymbol{y}_j^{(c)T}\boldsymbol{y}_j^{(c)}}{2\sigma^2_{\epsilon^{(c)}}} + \frac{1}{\sigma^2_{\epsilon^{(c)}}}\sum_{i\in R(j)}\mathbb{E}_q\Big[\gamma_{j,i}^{(c)}\beta_{j,i}^{(c)}\Big]\boldsymbol{X}_i^{(c)T}\boldsymbol{y}_j^{(c)} \\ &- \frac{1}{2\sigma^2_{\epsilon^{(c)}}}\sum_{i\in R(j)}\sum_{i'\neq i}\mathbb{E}_q\Big[(\gamma_{j,i'}^{(c)}\beta_{j,i'}^{(c)})(\gamma_{j,i}^{(c)}\beta_{j,i}^{(c)})\Big]\boldsymbol{X}_{i'}^{(c)T}\boldsymbol{X}_i^{(c)} \\ &- \frac{1}{2\sigma^2_{\epsilon^{(c)}}}\sum_{i\in R(j)}\mathbb{E}_q\Big[(\gamma_{j,i}^{(c)}\beta_{j,i}^{(c)})^2\Big]\boldsymbol{X}_i^{(c)T}\boldsymbol{X}_i^{(c)}\Big\} \\ &+ \sum_{c=1}^{C}\sum_{i\in R(j)}\Big(\frac{-\log(2\pi\sigma^2_\beta)}{2} - \mathbb{E}_q\Big[\frac{\beta_{j,i}^{(c)2}}{2\sigma^2_\beta}\Big]\Big) \\ &+ \sum_{c=1}^{C}\sum_{i\in R(j)}\mathbb{E}_q\Big[\gamma_{j,i}^{(c)}\log(g(z_{j,i}^{(c)})) + (1-\gamma_{j,i}^{(c)})\log(1-g(z_{j,i}^{(c)}))\Big] \\ &+ \sum_{i\in R(j)} -\frac{1}{2}\mathbb{E}_q\Big[\log(2\pi|S|)\Big] - \frac{1}{2}\mathbb{E}_q\Big[(\boldsymbol{z}_{j,i}-\boldsymbol{m})^T\boldsymbol{S}^{-1}(\boldsymbol{z}_{j,i}-\boldsymbol{m})\Big] \\ &- \log(2^{np/2}|\boldsymbol{V}|^{n/2}\Gamma_p(n/2)) + \frac{n-p-1}{2}\mathbb{E}_q\Big[\log|\boldsymbol{S}^{-1}|\Big] - \frac{1}{2}\mathbb{E}_q\Big[\text{Tr}(\boldsymbol{V}^{-1}\boldsymbol{S}^{-1})\Big] \end{aligned}$$

$$\mathbb{E}_q[\log q(\boldsymbol{\gamma}_j, \boldsymbol{\beta}_j, \boldsymbol{z}_j, \boldsymbol{S}^{-1})] = \Big(\sum_{c=1}^{C}\sum_{i\in R(j)}\mathbb{E}_q\Big[\log q(\beta_{j,i}^{(c)}|\gamma_{j,i}^{(c)})\Big] + \mathbb{E}_q\Big[\log q(\gamma_{j,i}^{(c)})\Big]\Big) + \Big(\sum_{i\in R(j)}\mathbb{E}_q\Big[\log q(\boldsymbol{z}_{j,i})\Big]\Big)$$

3.2.2 Expectation Terms

From the above expression of the ELBO, we observe that there are a number of expectation terms which we will need to calculate in order to calculate the ELBO. We write out this set of expectation equations, which will be useful for explicitly writing out the form of the ELBO.

$$\mathbb{E}_q[\gamma_{j,i}^{(c)}] = \alpha_{j,i}^{(c)}$$

$$\begin{aligned}\mathbb{E}_q[\gamma_{j,i}^{(c)}\beta_{j,i}^{(c)}] &= \sum_{\gamma_{j,i}^{(c)}} \int_{\beta_{j,i}^{(c)}} \gamma_{j,i}^{(c)}\beta_{j,i}^{(c)} q(\beta_{j,i}^{(c)}|\gamma_{j,i}^{(c)}) q(\gamma_{j,i}^{(c)}) d\beta_{j,i}^{(c)} \\ &= \int_{\beta_{j,i}^{(c)}} \beta_{j,i}^{(c)}\alpha_{j,i}^{(c)}\mathcal{N}(\beta_{j,i}^{(c)}|\mu_{j,i}^{(c)}, s_{j,i}^{(c)2}) d\beta_{j,i}^{(c)} = \alpha_{j,i}^{(c)}\mu_{j,i}^{(c)}\end{aligned}$$

$$\begin{aligned}\mathbb{E}_q[(\gamma_{j,i}^{(c)}\beta_{j,i}^{(c)})^2] &= \sum_{\gamma_{j,i}^{(c)}} \int_{\beta_{j,i}^{(c)}} \gamma_{j,i}^{(c)2}\beta_{j,i}^{(c)2} q(\beta_{j,i}^{(c)}|\gamma_{j,i}^{(c)}) q(\gamma_{j,i}^{(c)}) d\beta_{j,i}^{(c)} \\ &= \int_{\beta_{j,i}^{(c)}} \beta_{j,i}^{(c)2}\alpha_{j,i}^{(c)}\mathcal{N}(\beta_{j,i}^{(c)}|\mu_{j,i}^{(c)}, s_{j,i}^{(c)2}) d\beta_{j,i}^{(c)} = \alpha_{j,i}^{(c)}(s_{j,i}^{(c)2} + \mu_{j,i}^{(c)2})\end{aligned}$$

$$\begin{aligned}\mathbb{E}_q[\log q(\gamma_{j,i}^{(c)}, \beta_{j,i}^{(c)})] &= \sum_{\gamma_{j,i}^{(c)}} \int_{\beta_{j,i}^{(c)}} \log q(\gamma_{j,i}^{(c)}, \beta_{j,i}^{(c)}) q(\beta_{j,i}^{(c)}|\gamma_{j,i}^{(c)}) q(\gamma_{j,i}^{(c)}) d\beta_{j,i}^{(c)} \\ &= \alpha_{j,i}^{(c)}\log(\alpha_{j,i}^{(c)}) + (1-\alpha_{j,i}^{(c)})\log(1-\alpha_{j,i}^{(c)}) - \frac{1}{2}\Big(1 + \log(2\pi s_{j,i}^{(c)2})\Big)\end{aligned}$$

$$\mathbb{E}_q[\log q(\boldsymbol{z}_{j,i})] = \int_{\boldsymbol{z}_{j,i}} \Big(\log q(\boldsymbol{z}_{j,i})\Big) q(\boldsymbol{z}_{j,i}) d\boldsymbol{z}_{j,i} = -\frac{1}{2}\log|\tilde{\boldsymbol{S}}| - \frac{D}{2}(1 + \log(2\pi))$$

$$\begin{aligned}\mathbb{E}_q[\log p(\boldsymbol{z}_{j,i}|\boldsymbol{m}, \boldsymbol{S})] &= \mathbb{E}_q\Big[-\frac{1}{2}\log|\boldsymbol{S}| - \frac{1}{2}(\boldsymbol{z}_{j,i} - \boldsymbol{m})^T\boldsymbol{S}^{-1}(\boldsymbol{z}_{j,i} - \boldsymbol{m})\Big] \\ &= -\frac{1}{2}\log|\boldsymbol{S}| - \frac{1}{2}\mathbb{E}_q\Big[(\boldsymbol{z}_{j,i} - \boldsymbol{m})^T\boldsymbol{S}^{-1}(\boldsymbol{z}_{j,i} - \boldsymbol{m})\Big] \\ &= -\frac{1}{2}\log|\boldsymbol{S}| - \frac{1}{2}\mathrm{Tr}\Big(\boldsymbol{S}^{-1}\mathbb{E}_q\Big[(\boldsymbol{z}_{j,i} - \boldsymbol{m})^T(\boldsymbol{z}_{j,i} - \boldsymbol{m})\Big]\Big) \\ &= -\frac{1}{2}\log|\boldsymbol{S}| - \frac{1}{2}\mathrm{Tr}\Big(\boldsymbol{S}^{-1}\mathbb{E}_q\Big[(\boldsymbol{z}_{j,i} - \boldsymbol{m})(\boldsymbol{z}_{j,i} - \boldsymbol{m})^T\Big]\Big) \\ &= -\frac{1}{2}\log|\boldsymbol{S}| - \frac{1}{2}\mathrm{Tr}\Big(\boldsymbol{S}^{-1}\mathbb{E}_q\Big[\boldsymbol{z}_{j,i}\boldsymbol{z}_{j,i}^T - \boldsymbol{z}_{j,i}\boldsymbol{m}^T - \boldsymbol{m}\boldsymbol{z}_{j,i}^T + \boldsymbol{m}\boldsymbol{m}^T\Big]\Big) \\ &= -\frac{1}{2}\log|\boldsymbol{S}| - \frac{1}{2}\mathrm{Tr}\Big(\boldsymbol{S}^{-1}\Big(\tilde{\boldsymbol{m}}_{j,i}\tilde{\boldsymbol{m}}_{j,i}^T + \tilde{\boldsymbol{S}}_{j,i} - \tilde{\boldsymbol{m}}_{j,i}\boldsymbol{m}^T - \boldsymbol{m}\tilde{\boldsymbol{m}}_{j,i}^T + \boldsymbol{m}\boldsymbol{m}^T\Big)\Big) \\ &= -\frac{1}{2}\log|\boldsymbol{S}| - \frac{D}{2}\log(2\pi) - \frac{1}{2}\Big((\tilde{\boldsymbol{m}} - \boldsymbol{m})^T\boldsymbol{S}^{-1}(\tilde{\boldsymbol{m}}_{j,i} - \boldsymbol{m}) + \mathrm{Tr}(\boldsymbol{S}^{-1}\tilde{\boldsymbol{S}}_{j,i})\Big)\end{aligned}$$

$$\mathbb{E}_q[(\boldsymbol{z}_{j,i} - \boldsymbol{m})^T\boldsymbol{S}^{-1}(\boldsymbol{z}_{j,i} - \boldsymbol{m})] = (\tilde{\boldsymbol{m}}_{j,i} - \boldsymbol{m})^T\boldsymbol{S}^{-1}(\tilde{\boldsymbol{m}}_{j,i} - \boldsymbol{m}) + \mathrm{Tr}(\boldsymbol{S}^{-1}\tilde{\boldsymbol{S}}_{j,i})$$

$$\begin{aligned}\mathbb{E}_q[\log p(\boldsymbol{S}^{-1})] &= \mathbb{E}_q\Big[-\log(2^{np/2}|\boldsymbol{V}|^{n/2}\Gamma_p(n/2)) + \frac{n-p-1}{2}\log|\boldsymbol{S}^{-1}| - \frac{1}{2}\mathrm{Tr}(\boldsymbol{V}^{-1}\boldsymbol{S}^{-1})\Big] \\ &= -\log(2^{np/2}|\boldsymbol{V}|^{n/2}\Gamma_p(n/2)) + \frac{n-p-1}{2}\log|\boldsymbol{S}^{-1}| - \frac{1}{2}\mathrm{Tr}(\boldsymbol{V}^{-1}\boldsymbol{S}^{-1})\end{aligned}$$

3.2.3 Approximating the Expectation of Log Sigmoid

Here, we seek to approximate $\mathbb{E}_q[\log g(z_{j,i}^{(c)})]$ by first defining a grid of samples from the factor in our variational distribution that approximates the distribution for $\boldsymbol{z}_{j,i}$. In our current setup, this factor corresponds to a multivariate Gaussian centered at $\tilde{\boldsymbol{m}}_{j,i}$ with covariance $\tilde{\boldsymbol{S}}_{j,i}$. Because we are focusing on approximating a single scalar value $\mathbb{E}_q[\log g(z_{j,i}^{(c)})]$ here, we need only to consider the components of the multivariate Gaussian that directly correspond to element (c), namely $\tilde{m}_{j,i}^{(c)}$ in the mean and $\tilde{S}_{j,i}^{c,c}$ in the covariance. We refer to these values as $\tilde{m}$ and $\tilde{s}$, respectively, below for simplicity.

We choose a grid of samples $\tilde{m}$, $\tilde{m} \pm \eta\sqrt{\tilde{s}}$, $\tilde{m} \pm 2\eta\sqrt{\tilde{s}}$, ..., $\tilde{m} \pm K\eta\sqrt{\tilde{s}}$, where η and K are hyperparameters. Using this grid of samples, we approximate our expectation term as $\mathbb{E}_q[\log g(z_{j,i}^{(c)})] \approx \sum_{t=-K}^{K} w_t \log(g(\tilde{m} + t\eta\sqrt{\tilde{s}}))$, where $w_t = \Phi(t+\frac{1}{2}) - \Phi(t-\frac{1}{2})$.

Similarly, we approximate $\mathbb{E}_q[\log(1-g(z_{j,i}^{(c)}))]$ as $\mathbb{E}_q[\log(1-g(z_{j,i}^{(c)}))] \approx \sum_{t=-K}^{K} w_t \log(1-g(\tilde{m}+t\eta\sqrt{\tilde{s}}))$. With these approximations, let us now write out the ELBO.

$$
\begin{aligned}
\mathcal{L}(q) = \sum_{c=1}^{C} \Bigg\{ & -\frac{N^{(c)}}{2}\log(2\pi\sigma_\epsilon) - \frac{\boldsymbol{y}_j^{(c)T}\boldsymbol{y}^{(c)}}{2\sigma_\epsilon^2} + \frac{1}{\sigma_\epsilon^2}\sum_{i\in R(j)} \alpha_{j,i}^{(c)}\mu_{j,i}^{(c)}\boldsymbol{X}_i^{(c)T}\boldsymbol{y}_j^{(c)} \\
& - \frac{1}{2\sigma_{\epsilon^{(c)}}^2}\sum_{i\in R(j)}\sum_{i'\neq i}(\alpha_{j,i'}^{(c)}\mu_{j,i'}^{(c)})(\alpha_{j,i}^{(c)}\mu_{j,i}^{(c)})\boldsymbol{X}_{i'}^{(c)T}\boldsymbol{X}_i^{(c)} \\
& - \frac{1}{2\sigma_{\epsilon^{(c)}}^2}\sum_{i\in R(j)}\alpha_{j,i}^{(c)}(s_{j,i}^{(c)2}+\mu_{j,i}^{(c)2})\boldsymbol{X}_i^{(c)T}\boldsymbol{X}_i^{(c)} \Bigg\} \\
& + \sum_{c=1}^{C}\sum_{i\in R(j)}\alpha_{j,i}^{(c)}\left(\frac{-\log(2\pi\sigma_\beta^2)}{2} - \frac{s_{j,i}^{(c)2}+\mu_{j,i}^{(c)2}}{2\sigma_\beta^2}\right) \\
& + \sum_{c=1}^{C}\sum_{i\in R(j)}\alpha_{j,i}^{(c)}\left(\sum_{t=-K}^{K} w_t\log\left(g\left(\tilde{m}_{j,i}^{(c)} + t\eta\sqrt{\tilde{S}_{j,i}^{c,c}}\right)\right)\right) + (1-\alpha_{j,i}^{(c)})\left(\sum_{t=-K}^{K} w_t \log\left(1-g\left(\tilde{m}_{j,i}^{(c)}+t\eta\sqrt{\tilde{S}_{j,i}^{c,c}}\right)\right)\right) \\
& + \sum_{i\in R(j)} -\frac{1}{2}\left(-\psi_p(n/2) - p\log(2) - \log|\boldsymbol{S}^{-1}|\right) - \frac{D}{2}\log(2\pi) - \frac{1}{2}\left((\tilde{\boldsymbol{m}}_{j,i}-\boldsymbol{m})^T\boldsymbol{S}^{-1}(\tilde{\boldsymbol{m}}_{j,i}-\boldsymbol{m}) + \mathrm{Tr}(\boldsymbol{S}^{-1}\tilde{\boldsymbol{S}}_{j,i})\right) \\
& + \sum_{c=1}^{C}\sum_{i\in R(j)} -\alpha_{j,i}^{(c)}\log(\alpha_{j,i}^{(c)}) - (1-\alpha_{j,i}^{(c)})\log(1-\alpha_{j,i}^{(c)}) + \frac{1}{2}\left(1+\log(2\pi s_{j,i}^{(c)2})\right) \\
& + \sum_{i\in R(j)}\frac{1}{2}\log|\tilde{\boldsymbol{S}}_{j,i}| + \frac{D}{2}(1+\log(2\pi)) \\
& - \log(2^{np/2}|\boldsymbol{V}|^{n/2}\Gamma_p(n/2)) + \frac{n-p-1}{2}\log|\boldsymbol{S}^{-1}| - \frac{1}{2}\mathrm{Tr}(\boldsymbol{V}^{-1}\boldsymbol{S}^{-1})
\end{aligned}
$$

3.2.4 Coordinate ascent variational inference: $\tilde{\boldsymbol{S}}_{j,i}$

Let us first write out the terms in the ELBO that contain $\tilde{\boldsymbol{S}}_{j,i}$ or any of its elements.

$$\mathcal{L}(q) = k + \sum_{c=1}^{C} \sum_{i \in R(j)} \alpha_{j,i}^{(c)} \Bigg(\sum_{t=-K}^{K} w_t \log \bigg(g\bigg(\tilde{m}_{j,i}^{(c)} + t\eta\sqrt{\tilde{S}_{j,i}^{c,c}} \bigg) \bigg) \Bigg) + (1-\alpha_{j,i}^{(c)}) \Bigg(\sum_{t=-K}^{K} w_t \log \bigg(1 - g\bigg(\tilde{m}_{j,i}^{(c)} + t\eta\sqrt{\tilde{S}_{j,i}^{c,c}} \bigg) \bigg) \Bigg)$$
$$+ \sum_{i \in R(j)} \frac{1}{2} \log |\tilde{\boldsymbol{S}_{j,i}}| - \frac{D}{2}\log(2\pi) - \frac{1}{2}\Big((\tilde{\boldsymbol{m}}_{j,i} - \boldsymbol{m})^T \boldsymbol{S}^{-1} (\tilde{\boldsymbol{m}}_{j,i} - \boldsymbol{m}) + \mathrm{Tr}(\boldsymbol{S}^{-1}\tilde{\boldsymbol{S}}_{j,i}) \Big)$$

Now, we take the partial derivative with respect to $\tilde{\boldsymbol{S}}_{j,i}$.

$$\frac{\partial \mathcal{L}}{\partial \tilde{\boldsymbol{S}}_{j,i}} = \frac{\partial}{\partial \tilde{\boldsymbol{S}}_{j,i}} \Bigg(\alpha_{j,i}^{(c)} \Bigg(\sum_{t=-K}^{K} w_t \log \bigg(g\bigg(\tilde{m}_{j,i}^{(c)} + t\eta\sqrt{\tilde{S}_{j,i}^{c,c}} \bigg) \bigg) \Bigg) + (1-\alpha_{j,i}^{(c)}) \Bigg(\sum_{t=-K}^{K} w_t \log \bigg(1 - g\bigg(\tilde{m}_{j,i}^{(c)} + t\eta\sqrt{\tilde{S}_{j,i}^{c,c}} \bigg) \bigg) \Bigg) \Bigg)$$
$$- \frac{1}{2}\boldsymbol{S}^{-1} + \frac{1}{2}\tilde{\boldsymbol{S}}_{j,i}^{-1}$$

We simplify each of the terms in the first line to clean up the calculation of the partial derivative, replacing $\tilde{S}_{j,i}^{c,c}$ with s and making the following substitutions: $a = e^{-\tilde{m}_{j,i}^{(c)}}$ and $k_t = \eta t$. We also apply the sigmoid function $g(x) = \frac{1}{1+e^{-x}}$.

$$\frac{\partial}{\partial s}\Bigg(\log \frac{1}{1+e^{-(\tilde{m}_{j,i}^{(c)} + \eta t\sqrt{s})}} \Bigg) = \frac{\partial}{\partial s}\Bigg(\log \frac{1}{1+(e^{-\tilde{m}_{j,i}^{(c)}})(e^{-\eta t\sqrt{s}})} \Bigg) = \frac{\partial}{\partial s}\Bigg(\log \frac{1}{1+ae^{-ks}} \Bigg)$$
$$= \frac{k}{2\sqrt{s}}\Bigg(1 - \frac{1}{1+ae^{-k\sqrt{s}}} \Bigg) = \frac{k}{2\sqrt{s}}\Bigg(1 - \frac{1}{1+e^{-(\tilde{m}_{j,i}^{(c)} + \eta t\sqrt{s})}} \Bigg) = \frac{\eta t}{2\sqrt{s}}\Big(1 - g(\tilde{m}_{j,i}^{(c)} + \eta t\sqrt{s}) \Big)$$

$$\frac{\partial}{\partial s}\Bigg(\log \bigg(1 - \frac{1}{1+e^{-(\tilde{m}_{j,i}^{(c)} + \eta t\sqrt{s})}} \bigg) \Bigg) = \frac{\partial}{\partial s}\Bigg(\log \bigg(1 - \frac{1}{1+(e^{-\tilde{m}_{j,i}^{(c)}})(e^{-\eta t\sqrt{s}})} \bigg) \Bigg) = \frac{\partial}{\partial s}\Bigg(\log(1 - \frac{1}{1+ae^{-k\sqrt{s}}}) \Bigg)$$
$$= -\frac{k}{2\sqrt{s}} \frac{1}{1+ae^{-k\sqrt{s}}} = \frac{-\eta t}{2\sqrt{s}} \frac{1}{1+e^{-(\tilde{m}_{j,i}^{(c)} + \eta t\sqrt{s})}} = -\frac{-\eta t}{2\sqrt{s}} g(\tilde{m}_{j,i}^{(c)} + \eta t\sqrt{s})$$

Substituting these terms back into our expression for $\frac{\partial \mathcal{L}}{\partial \tilde{\boldsymbol{S}}_{j,i}}$ yields the following.

$$\frac{\partial \mathcal{L}}{\partial \tilde{\boldsymbol{S}}_{j,i}} = -\frac{1}{2}\boldsymbol{S}^{-1} + \frac{1}{2}\tilde{\boldsymbol{S}}_{j,i}^{-1} + \boldsymbol{D}$$

Here, $\boldsymbol{D} = \mathrm{diag}(d^{(1)}, ..., d^{(C)})$, where

$$d^{(c)} = \sum_{t=-K}^{K} \Bigg(\alpha_{j,i}^{(c)} w_t \frac{\eta t}{2\sqrt{s}} \bigg(1 - \frac{1}{1+e^{-(\tilde{m}_{j,i}^{(c)} + \eta ts)}} \bigg) + (1-\alpha_{j,i}^{(c)}) w_t \frac{-\eta t}{2\sqrt{s}} \bigg(\frac{1}{1+e^{-(\tilde{m}_{j,i}^{(c)} + \eta ts)}} \bigg) \Bigg)$$
$$= \sum_{t=-K}^{K} \Bigg(\alpha_{j,i}^{(c)} w_t \frac{\eta t}{2\sqrt{s}} \Big(1 - g(\tilde{m}_{j,i}^{(c)} + \eta t\sqrt{s}) \Big) - (1-\alpha_{j,i}^{(c)})(w_t \frac{\eta t}{2\sqrt{s}}) g(\tilde{m}_{j,i}^{(c)} + \eta t\sqrt{s}) \Bigg)$$
$$= \sum_{t=-K}^{K} w_t \frac{\eta t}{2\sqrt{s}} \Bigg(\alpha_{j,i}^{(c)} \Big(1 - g(\tilde{m}_{j,i}^{(c)} + \eta t\sqrt{s}) \Big) - (1-\alpha_{j,i}^{(c)}) \Big(g(\tilde{m}_{j,i}^{(c)} + \eta t\sqrt{s}) \Big) \Bigg)$$
$$= \sum_{t=-K}^{K} w_t \frac{\eta t}{2\sqrt{s}} \Big(\alpha_{j,i}^{(c)} - \alpha_{j,i}^{(c)} g(\tilde{m}_{j,i}^{(c)} + \eta ts) - g(\tilde{m}_{j,i}^{(c)} + \eta t\sqrt{s}) + \alpha_{j,i}^{(c)} g(\tilde{m}_{j,i}^{(c)} + \eta t\sqrt{s}) \Big)$$

$$= \sum_{t=-K}^{K} \frac{w_t \eta t}{2\sqrt{s}} \left(\alpha_{j,i}^{(c)} - g\left(\tilde{m}_{j,i}^{(c)} + \eta t \sqrt{s} \right) \right) = \sum_{t=-K}^{K} \frac{w_t \eta t}{2\sqrt{\tilde{S}_{j,i}^{c,c}}} \left(\alpha_{j,i}^{(c)} - g\left(\tilde{m}_{j,i}^{(c)} + \eta t \sqrt{\tilde{S}_{j,i}^{c,c}} \right) \right)$$

Now that we have an expression for the gradient, we can use gradient ascent to optimize $\tilde{S}_{j,i}^{c,c}$.

3.2.5 Coordinate ascent variational inference: $\tilde{m}_{j,i}^{(c)}$

Similar to before, we start out by isolating the terms in the ELBO that contain $\tilde{\boldsymbol{m}}_{j,i}$.

$$\mathcal{L}(q) = k + \sum_{c=1}^{C} \sum_{i \in R(j)} \alpha_{j,i}^{(c)} \left(\sum_{t=-K}^{K} w_t \log \left(g\left(\tilde{m}_{j,i}^{(c)} + t\eta \sqrt{\tilde{S}_{j,i}^{c,c}} \right) \right) \right) + (1 - \alpha_{j,i}^{(c)}) \left(\sum_{t=-K}^{K} w_t \log \left(1 - g\left(\tilde{m}_{j,i}^{(c)} + t\eta \sqrt{\tilde{S}_{j,i}^{c,c}} \right) \right) \right)$$
$$+ \sum_{i \in R(j)} -\frac{1}{2} \log |\boldsymbol{S}| - \frac{D}{2} \log(2\pi) - \frac{1}{2} \left((\tilde{\boldsymbol{m}}_{j,i} - \boldsymbol{m})^T \boldsymbol{S}^{-1} (\tilde{\boldsymbol{m}}_{j,i} - \boldsymbol{m}) + \mathrm{Tr}(\boldsymbol{S}^{-1} \tilde{\boldsymbol{S}}_{j,i}) \right)$$

Taking the partial derivative with respect to $\tilde{m}_{j,i}$, we get the following.

$$\frac{\partial \mathcal{L}}{\partial \tilde{m}_{j,i}^{(c)}} = \frac{\partial}{\partial \tilde{m}_{j,i}} \left(\alpha_{j,i}^{(c)} \left(\sum_{t=-K}^{K} w_t \log \left(g\left(\tilde{m}_{j,i}^{(c)} + t\eta \sqrt{\tilde{S}_{j,i}^{c,c}} \right) \right) \right) + (1 - \alpha_{j,i}^{(c)}) \left(\sum_{t=-K}^{K} w_t \log \left(1 - g\left(\tilde{m}_{j,i}^{(c)} + t\eta \sqrt{\tilde{S}_{j,i}^{c,c}} \right) \right) \right) \right)$$
$$+ \sum_{c'=1}^{C} -(\tilde{m}_{j,i}^{(c')} - m^{(c')}) \boldsymbol{S}_{c,c'}^{-1} = 0$$

We then calculate the remaining partial derivative terms in the equation above.

$$\frac{\partial}{\partial \tilde{m}_{j,i}^{(c)}} \left(\log \frac{1}{1 + e^{-(\tilde{m}_{j,i}^{(c)} + \eta t \sqrt{\tilde{S}_{j,i}^{c,c}})}} \right) = 1 - \frac{1}{1 + e^{-(\tilde{m}_{j,i}^{(c)} + \eta t \sqrt{\tilde{S}_{j,i}^{c,c}})}} = 1 - g\left(\tilde{m}_{j,i}^{(c)} + \eta t \sqrt{\tilde{S}_{j,i}^{c,c}} \right)$$

$$\frac{\partial}{\partial \tilde{m}_{j,i}^{(c)}} \left(\log 1 - \frac{1}{1 + e^{-(\tilde{m}_{j,i}^{(c)} + \eta t \sqrt{\tilde{S}_{j,i}^{c,c}})}} \right) = \frac{-1}{1 + e^{-(\tilde{m}_{j,i}^{(c)} + \eta t \sqrt{\tilde{S}_{j,i}^{c,c}})}} = -g\left(\tilde{m}_{j,i}^{(c)} + \eta t \sqrt{\tilde{S}_{j,i}^{c,c}} \right)$$

Our expression for the partial derivative can now be written as follows.

$$\frac{\partial \mathcal{L}}{\partial \tilde{m}_{j,i}} = \alpha_{j,i}^{(c)} \left(\sum_{t=-K}^{K} w_t \left(1 - \frac{1}{1 + e^{-(\tilde{m}_{j,i}^{(c)} + \eta t \sqrt{\tilde{S}_{j,i}^{c,c}})}} \right) \right) + (1 - \alpha_{j,i}^{(c)}) \left(\sum_{t=-K}^{K} w_t \frac{-1}{1 + e^{-(\tilde{m}_{j,i}^{(c)} + \eta t \sqrt{\tilde{S}_{j,i}^{c,c}})}} \right)$$
$$+ \sum_{c'=1}^{C} -(\tilde{m}_{j,i}^{(c')} - m^{(c')}) \boldsymbol{S}_{c,c'}^{-1}$$
$$= \alpha_{j,i}^{(c)} \sum_{t=-K}^{K} w_t \left(1 - g\left(\tilde{m}_{j,i}^{(c)} + \eta t \sqrt{\tilde{S}_{j,i}^{c,c}} \right) \right) - (1 - \alpha_{j,i}^{(c)}) \left(\sum_{t=-K}^{K} w_t g\left(\tilde{m}_{j,i}^{(c)} + \eta t \sqrt{\tilde{S}_{j,i}^{c,c}} \right) \right)$$
$$+ \sum_{c'=1}^{C} -(\tilde{m}_{j,i}^{(c')} - m^{(c')}) \boldsymbol{S}_{c,c'}^{-1}$$
$$= \sum_{t=-K}^{K} w_t \left(\alpha_{j,i}^{(c)} \left(1 - g\left(\tilde{m}_{j,i}^{(c)} + \eta t \sqrt{\tilde{S}_{j,i}^{c,c}} \right) \right) - (1 - \alpha_{j,i}^{(c)}) g\left(\tilde{m}_{j,i}^{(c)} + \eta t \sqrt{\tilde{S}_{j,i}^{c,c}} \right) \right)$$
$$+ \sum_{c'=1}^{C} -(\tilde{m}_{j,i}^{(c')} - m^{(c')}) \boldsymbol{S}_{c,c'}^{-1}$$

$$= \sum_{t=-K}^{K} w_t \left(\alpha_{j,i}^{(c)} - g\left(\tilde{m}_{j,i}^{(c)} + \eta t \sqrt{\tilde{S}_{j,i}^{c,c}} \right) \right) + \sum_{c'=1}^{C} -(\tilde{m}_{j,i}^{(c')} - m^{(c')}) \boldsymbol{S}_{c,c'}^{-1} = 0$$

As with the optimization scheme for $\tilde{\boldsymbol{S}}_{j,i}$, we can employ this expression of the gradient in a gradient ascent optimization procedure to identify an optimal value for $\tilde{m}_{j,i}$.

3.2.6 Coordinate ascent variational inference: $s_{j,i}^{(c)2}$

$$\begin{aligned}
\frac{\partial \mathcal{L}}{\partial s_{j,i}^{(c)2}} &= -\frac{1}{2\sigma_\epsilon^2} \alpha_{j,i}^{(c)} (\boldsymbol{X}_i^{(c)T} \boldsymbol{X}_i^{(c)}) - \frac{1}{2\sigma_\beta^2} + \frac{1}{2 s_{j,i}^{(c)}} = 0 \\
\frac{1}{s_{j,i}^{(c)2}} &= \frac{1}{\sigma_\beta^2} (\boldsymbol{X}_i^{(c)T} \boldsymbol{X}_i^{(c)}) + \frac{1}{\sigma_\beta^2} \\
s_{j,i}^{(c)2} &= \frac{1}{\frac{1}{\sigma_\epsilon^2} \alpha_{j,i}^{(c)} (\boldsymbol{X}_i^{(c)T} \boldsymbol{X}_i^{(c)}) + \frac{1}{\sigma_\beta^2}} = \frac{\sigma_\epsilon^2}{\alpha_{j,i}^{(c)} (\boldsymbol{X}_i^{(c)T} \boldsymbol{X}_i^{(c)}) + \frac{\sigma_\epsilon^2}{\sigma_\beta^2}}
\end{aligned}$$

3.2.7 Coordinate ascent variational inference: $\mu_{j,i}^{(c)}$

$$\begin{aligned}
\frac{\partial \mathcal{L}}{\partial \mu_{j,i}^{(c)}} &= \frac{1}{\sigma_\epsilon^2} \alpha_{j,i}^{(c)} \boldsymbol{X}_i^{(c)} y^{(c)} - \frac{1}{\sigma_\epsilon^2} (\boldsymbol{X}_i^{(c)T} \boldsymbol{X}_i^{(c)}) \alpha_{j,i}^{(c)} \mu_{j,i}^{(c)} - \frac{1}{\sigma_\epsilon^2} \sum_{i' \neq i} (\boldsymbol{X}_i^{(c)T} \boldsymbol{X}_{i'}^{(c)}) \alpha_{j,i'}^{(c)} \mu_{j,i'}^{(c)} \alpha_{j,i}^{(c)} - \frac{1}{\sigma_\beta^2} \mu_{j,i}^{(c)} \\
\frac{1}{\sigma_\epsilon^2} \alpha_{j,i}^{(c)} \boldsymbol{X}_i^{(c)} y^{(c)} - \frac{1}{\sigma_\epsilon^2} \sum_{i' \neq i} (\boldsymbol{X}_i^{(c)T} \boldsymbol{X}_{i'}^{(c)}) \alpha_{j,i'}^{(c)} \mu_{j,i'}^{(c)} \alpha_{j,i}^{(c)} &= \mu_{j,i}^{(c)} \left(\frac{1}{\sigma_\beta^2} + \frac{1}{\sigma_\epsilon^2} \alpha_{j,i}^{(c)} \boldsymbol{X}_i^{(c)T} \boldsymbol{X}_i^{(c)} \right) \\
\frac{\alpha_{j,i}^{(c)}}{\sigma_\epsilon^2} \left(X_i^{(c)} y^{(c)} - \frac{1}{\sigma_\epsilon^2} \sum_{i' \neq i} (\boldsymbol{X}_i^{(c)T} \boldsymbol{X}_{i'}^{(c)}) \alpha_{j,i'}^{(c)} \mu_{j,i'}^{(c)} \right) &= \mu_{j,i}^{(c)} \left(\frac{1}{s_{j,i}^{(c)2}} \right) \\
\mu_{j,i}^{(c)} &= \frac{\alpha_{j,i}^{(c)} s_{j,i}^{(c)2}}{\sigma_\epsilon^2} \left(\boldsymbol{X}_i^{(c)} y^{(c)} - \sum_{i' \neq i} \alpha_{j,i'}^{(c)} \mu_{j,i'}^{(c)} (\boldsymbol{X}_i^{(c)T} \boldsymbol{X}_{i'}^{(c)}) \right)
\end{aligned}$$

3.2.8 Coordinate ascent variational inference: $\alpha_{j,i}^{(c)}$

$$\begin{aligned}
\frac{\partial \mathcal{L}}{\partial \alpha_{j,i}^{(c)}} &= \frac{1}{\sigma_\epsilon^2} \mu_{j,i}^{(c)} \boldsymbol{X}_i^{(c)} \boldsymbol{y}^{(c)} - \frac{1}{\sigma_\epsilon^2} \sum_{i' \neq i} \alpha_{j,i'}^{(c)} \mu_{j,i'}^{(c)} \mu_{j,i}^{(c)} \boldsymbol{X}_{i'}^{(c)T} \boldsymbol{X}_i^{(c)} \\
&\quad - \frac{1}{2\sigma_\epsilon^2} (s_{j,i}^{(c)2} + \mu_{j,i}^{(c)2}) \boldsymbol{X}_i^{(c)T} \boldsymbol{X}_i^{(c)} \\
&\quad + \left(\sum_{t=-K}^{K} w_t \log \left(g\left(\tilde{m}_{j,i}^{(c)} + t\eta \sqrt{\tilde{S}_{j,i}^{c,c}} \right) \right) \right) - \left(\sum_{t=-K}^{K} w_t \log \left(1 - g\left(\tilde{m}_{j,i}^{(c)} + t\eta \sqrt{\tilde{S}_{j,i}^{c,c}} \right) \right) \right) \\
&\quad - (1 + \log(\alpha_{j,i}^{(c)})) + (\log(1 - \alpha_{j,i}^{(c)}) + 1) \\
&= u_{j,i}^{(c)} - (1 + \log(\alpha_{j,i}^{(c)})) + (\log(1 - \alpha_{j,i}^{(c)}) + 1) \\
&= u_{j,i}^{(c)} + \log(\frac{1 - \alpha_{j,i}^{(c)}}{\alpha_{j,i}^{(c)}}) = 0 \\
&\Rightarrow \alpha_{j,i}^{(c)} = \frac{1}{1 + e^{-u_{j,i}^{(c)}}}
\end{aligned}$$

$$u_{j,i}^{(c)} = \frac{1}{\sigma_\epsilon^2} \mu_{j,i}^{(c)} \boldsymbol{X}_i^{(c)} \boldsymbol{y}^{(c)} - \frac{1}{\sigma_\epsilon^2} \sum_{i' \neq i} \alpha_{j,i'}^{(c)} \mu_{j,i'}^{(c)} \mu_{j,i}^{(c)} \boldsymbol{X}_{i'}^{(c)T} \boldsymbol{X}_i^{(c)}$$

$$
\begin{aligned}
&\quad - \frac{1}{2\sigma_\epsilon^2}(s_{j,i}^{(c)2} + \mu_{j,i}^{(c)2})\boldsymbol{X}_i^{(c)T}\boldsymbol{X}_i^{(c)} + b + 2\sum_{c' \in N(c)} W_{c,c'}(2\alpha_{j,i}^{(c')} - 1) \\
&= \frac{\mu_{j,i}^{(c)}}{\sigma_\epsilon^2}\left(\boldsymbol{X}_i^{(c)}\boldsymbol{y}^{(c)} - \sum_{i' \neq i}\alpha_{j,i'}^{(c)}\mu_{j,i'}^{(c)}\boldsymbol{X}_{i'}^{(c)T}\boldsymbol{X}_i^{(c)}\right) - \frac{1}{2\sigma_\epsilon^2}(s_{j,i}^{(c)2} + \mu_{j,i}^{(c)2})\boldsymbol{X}_i^{(c)T}\boldsymbol{X}_i^{(c)} \\
&\quad + \left(\sum_{t=-K}^{K} w_t \log\left(g\left(\tilde{m}_{j,i}^{(c)} + t\eta\sqrt{\tilde{S}_{j,i}^{c,c}}\right)\right)\right) - \left(\sum_{t=-K}^{K} w_t \log\left(1 - g\left(\tilde{m}_{j,i}^{(c)} + t\eta\sqrt{\tilde{S}_{j,i}^{c,c}}\right)\right)\right)
\end{aligned}
$$

4 Runtime and Memory Usage

ShareNet's runtime and memory usage are dependent on a variety of factors, most notably the number of cell types and the number of putative edges in a network. The main bottleneck for both of these computational resources is related to the $\tilde{\boldsymbol{S}}$ variational parameter, an $N \times C \times C$ matrix, where N is the number of putative edges in a network and C is the number of cell types. The memory associated with this parameter, therefore, scales with complexity $O(NC^2)$. Meanwhile, the matrix inversion step in the coordinate ascent update for $\tilde{\boldsymbol{S}}$ (Eq. 8) scales with complexity $O(NC^3)$. As a result, the number of network edges and cell types considered in the use of ShareNet can dramatically influence both the runtime and memory consumption, a phenomenon we observe across the three datasets, which spanned a broad range of these factors.

For the BEELINE analyses, we considered 267,750 putative edges for each of the five cell types in the dataset. In the Tabula muris analyses, we inferred networks for 52 cell types, each of which contained 2,640,456 putative edges. Lastly, the mouse blood lineage dataset contained 24 cell types, and each network was associated with 1,207,152 putative edges. The average total runtime for ShareNet was 2 minutes, 116 minutes, and 411 minutes for the BEELINE, mouse blood lineage, and Tabula muris datasets, respectively. Peak memory usage was approximately 664 MB, 11.9 GB, and 111 GB for the same three datasets. These runtime and memory usage statistics do not include runs of the network inference algorithms required to obtain initial network estimates and standard deviation estimates, which ShareNet uses as input. All methods were run on a 2.40GHz Intel Xeon E5-2695v2 central processing unit.

While the runtime and memory usage statistics may appear sizeable for larger datasets, we note that both of these computational resources can be substantially reduced by leveraging prior biological knowledge. For example, the number of putative edges considered in a network can be drastically reduced to consider only edges supported by transcription factor binding motifs or chromatin accessibility instead of all possible transcription factor-target gene pairs as was done in this study. In addition, we observed that cell types that are dissimilar tend to engage in less information sharing with each other in ShareNet (Fig. 5a, 6b). Retaining only cell types that are more similar to each other for use in ShareNet rather than using an entire collection of varied cell types could, therefore, also reduce the computational burden for such large dataset scenarios without diminishing performance.
